# Glymphatic system bridges peripheral and central nervous system changes in classic trigeminal neuralgia

**DOI:** 10.1093/braincomms/fcag191

**Published:** 2026-06-03

**Authors:** Jingqi Jiang, Pengfei Zhang, Fei Jia, Jun Wang, Liang Zhou, Zhuo Wang, Wenjing Huang, Shu Cui, Xiaohua Zhang, Luyang Zhang, Keshen Wang, Kai Ai, Laiyang Ma, Jing Zhang

**Affiliations:** Department of Magnetic Resonance, The Second Hospital & Clinical Medical School, Lanzhou University, Lanzhou, Gansu 730030, China; Gansu Province Clinical Research Center for Functional and Molecular Imaging, Lanzhou, Gansu 730030, China; Department of Radiology, West China Hospital of Sichuan University, Chengdu, Sichuan 610041, China; Department of Magnetic Resonance, The Second Hospital & Clinical Medical School, Lanzhou University, Lanzhou, Gansu 730030, China; Gansu Province Clinical Research Center for Functional and Molecular Imaging, Lanzhou, Gansu 730030, China; Department of Magnetic Resonance, The Second Hospital & Clinical Medical School, Lanzhou University, Lanzhou, Gansu 730030, China; Gansu Province Clinical Research Center for Functional and Molecular Imaging, Lanzhou, Gansu 730030, China; Department of Magnetic Resonance, The Second Hospital & Clinical Medical School, Lanzhou University, Lanzhou, Gansu 730030, China; Gansu Province Clinical Research Center for Functional and Molecular Imaging, Lanzhou, Gansu 730030, China; Department of Magnetic Resonance, The Second Hospital & Clinical Medical School, Lanzhou University, Lanzhou, Gansu 730030, China; Gansu Province Clinical Research Center for Functional and Molecular Imaging, Lanzhou, Gansu 730030, China; Department of Magnetic Resonance, The Second Hospital & Clinical Medical School, Lanzhou University, Lanzhou, Gansu 730030, China; Gansu Province Clinical Research Center for Functional and Molecular Imaging, Lanzhou, Gansu 730030, China; The Second Hospital & Clinical Medical School, Lanzhou University, Lanzhou, Gansu 730030, China; The Second Hospital & Clinical Medical School, Lanzhou University, Lanzhou, Gansu 730030, China; The Second Hospital & Clinical Medical School, Lanzhou University, Lanzhou, Gansu 730030, China; Department of Neurosurgery and Laboratory of Neurosurgery, The Second Hospital & Clinical Medical School, Lanzhou University, Lanzhou, Gansu 730030, China; Department of Clinical and Technical Supports, Philips Healthcare, Xi’an, Shanxi 710000, China; Department of Magnetic Resonance, The Second Hospital & Clinical Medical School, Lanzhou University, Lanzhou, Gansu 730030, China; Gansu Province Clinical Research Center for Functional and Molecular Imaging, Lanzhou, Gansu 730030, China; Department of Magnetic Resonance, The Second Hospital & Clinical Medical School, Lanzhou University, Lanzhou, Gansu 730030, China; Gansu Province Clinical Research Center for Functional and Molecular Imaging, Lanzhou, Gansu 730030, China

**Keywords:** classical trigeminal neuralgia, neurite orientation dispersion and density imaging, glymphatic system, neurovascular contact, tract-based spatial statistics

## Abstract

Classical trigeminal neuralgia exhibits both peripheral neurovascular compression and widespread central white matter alterations, but the mechanisms bridging peripheral and central pathology remain elusive. This study investigated the role of glymphatic system dysfunction in linking peripheral and central pathology. We prospectively enrolled 115 classical trigeminal neuralgia patients and 87 healthy controls, using advanced multi-shell diffusion MRI to assess microstructural integrity (via neurite orientation dispersion and density imaging metrics) and glymphatic function (via the diffusion tensor imaging along the perivascular space index). Results demonstrated significant microstructural alterations at neurovascular compression sites, including lower fractional anisotropy and neurite density index, along with a higher fraction of isotropic water in affected trigeminal nerves compared to the unaffected sides and healthy controls (*P_FDR_* < 0.050). The diffusion tensor imaging along the perivascular space index on the affected side was significantly lower than that on the unaffected side and that in healthy controls (*P_FDR_* < 0.050). Tract-based spatial statistics analyses revealed bilateral microstructural changes regardless of pain laterality, with distinct neural remodelling patterns observed in the left- and right-pain side groups (*P* < 0.050, family-wise error-corrected). We also found that higher fraction of isotropic water at the neurovascular compression site correlated with higher pain intensity (*P_FDR_* = 0.013), while reduced glymphatic function (both affected side and total diffusion tensor imaging along the perivascular space indexes) was associated with worse pain and psychological scores (*P_FDR_* < 0.050). Mediation analyses in right-pain side patients indicated that neurite density index reduction at the neurovascular compression site influenced widespread white matter neurite density index reduction through impaired glymphatic function. Diagnostic receiver operating characteristic analyses showed that individual imaging markers could discriminate classical trigeminal neuralgia, and integrated models combining peripheral metrics, affected side diffusion tensor imaging along the perivascular space indexes, and central metrics achieved superior diagnostic power. Our findings provide novel evidence that glymphatic dysfunction may be a key mechanism linking peripheral compression to global neural degeneration in classical trigeminal neuralgia, offering new imaging biomarkers for diagnosis and pathophysiological insight.

## Teaser

Jiang *et al*. report that classic trigeminal neuralgia involves peripheral nerve and brain changes. Impaired glymphatic function measured by diffusion tensor imaging along perivascular space index may connect these abnormalities. Imaging markers correlate with pain severity and emotional disorder, suggesting glymphatic dysfunction drives chronic pain, offering new clinical targets.

## Graphical Abstract

**Graphical Abstract:**
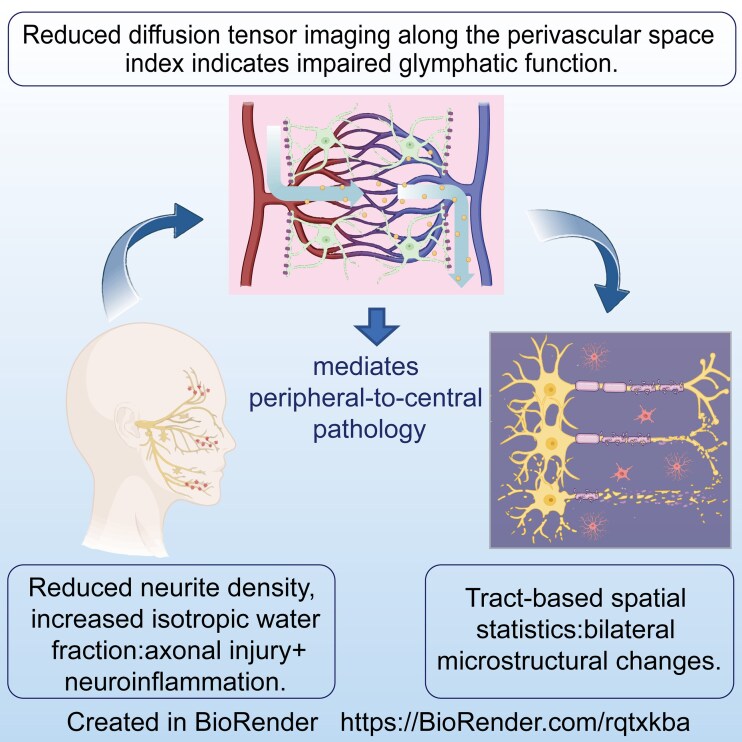

## Introduction

Classical trigeminal neuralgia (CTN) is a severe neuralgia disorder characterized by unilateral, paroxysmal, electric shock-like or stabbing facial pain confined to the distribution of the trigeminal nerve.^1^ The pathology of CTN often involves the central-peripheral transition zone of the trigeminal nerve root, a vulnerable region characterized by a weakened blood–nerve barrier that is particularly susceptible to neurovascular compression (NVC), leading to focal demyelination.^2^ However, it remains unclear how this peripheral demyelination transforms into persistent pain.

Animal studies have demonstrated that peripheral nerve injury can induce fibroblasts within the trigeminal ganglion to release the inflammatory cytokine interleukin-33. This, in turn, enhances neuronal excitability through the activation of tumorigenicity 2-transient receptor potential ankyrin 1 signalling pathway, leading to mechanical allodynia.^3^ This evidence underscores the pathological role of neuroinflammation in CTN. In human studies, diffusion tensor imaging (DTI) provides imaging evidence for this process: the lower fractional anisotropy (FA) (*P* < 0.010) and higher radial diffusivity (RD) (*P* < 0.050) at the site of NVC directly reflect demyelinating changes, while the higher mean diffusivity (MD) (*P* < 0.050) indicates the presence of local neuroinflammation.^4^ Notably, the impact of neuroinflammation is not limited to the NVC region. Zhao *et al*.^5^ observed not only demyelination in the axons of the infraorbital nerve in a TN rat model using electron microscopy but also identified ultrastructural alterations in other regions of the brain, suggesting that neuroinflammation may extend beyond peripheral nerves and involve the central nervous system (CNS). Liu *et al*.^6^ employed tract-based spatial statistics (TBSS) and multiple diffusion metrics for whole-brain voxel-wise analysis, revealing selective damage to extensive white matter (WM) fibre tracts in CTN patients, further supporting the existence of widespread brain WM alterations secondary to NVC.

These findings raise a critical question: how does peripheral nerve injury lead to global brain microstructural alterations? Emerging evidence implicates dysfunction of the glymphatic system (GS), a recently discovered waste clearance pathway in the brain, which has garnered increasing attention.^7^ Neuroinflammation may impair GS function, leading to the accumulation of toxic metabolites and further exacerbating neuronal damage, linking peripheral nerve injury to broader neural degradation.^8^ The diffusion tensor imaging analysis along the perivascular space (DTI-ALPS) index is a non-invasive MRI marker proposed to reflect glymphatic function. Recent research suggests that the DTI-ALPS index may serve as a biomarker for CNS involvement in CTN^9^. Based on this evidence, we hypothesize that alterations in the DTI-ALPS index in CTN patients mediate the progression from peripheral neuroinflammation to global brain microstructural damage.

However, conventional DTI lacks the specificity to fully characterize axonal and dendritic complexity. Neurite orientation dispersion and density imaging (NODDI), an advanced diffusion MRI technique, overcomes this limitation by quantifying the neurite density index (NDI) and orientation dispersion index (ODI), offering superior sensitivity to microstructural changes in both peripheral and central neural pathways^10^. In this study, we aim to test our hypothesis by employing NODDI to assess microstructural integrity at the site of NVC and across the brain, combined with the DTI-ALPS index to evaluate glymphatic function, and to address the following three questions: (i) Whether diffusion parameters at the NVC site and the DTI-ALPS index are altered in CTN patients; (ii) whether the microstructural integrity of the whole brain is disrupted in CTN patients and (iii) whether the DTI-ALPS index mediates the progression from peripheral nerve injury to global brain microstructural changes.

## Materials and methods

### Study participants

This study prospectively enrolled 115 patients who visited the neurosurgery department between February 2024 and October 2025. All patients were diagnosed with ‘CTN, purely paroxysmal’ according to the International Classification of Headache Disorders (ICHD-III) by two independent neurologists,^11^ corroborated by 3.0 T MRI to confirm NVC.^12^

Inclusion criteria were (i) age ≥18 years; (ii) unilateral CTN with a disease duration ≥ 6 months; (iii) paroxysmal, electric shock-like pain with identifiable trigger points and (iv) absence of significant sensory deficits.

Exclusion criteria were (i) persistent pain; (ii) history of cranial surgery (especially microvascular decompression (MVD) or other surgeries related to CTN); (iii) presence of any other chronic pain disorders or major neuropsychiatric conditions; (iv) metal implants in the body; (v) abnormal MRI findings (including severe WM lesions of Fazekas grade III, evidence of demyelinating diseases such as multiple sclerosis, or space-occupying lesions suggestive of secondary TN); (vi) left-handedness and (vii) inability to tolerate the MRI examination.^13^ The healthy controls (HCs) consisted of 87 volunteers matched to the CTN group in terms of age, sex and educational level. Demographic information was recorded for all participants. Pain intensity was assessed using the Visual Analog Scale (VAS), ranging from 0 (no pain) to 10 (the most severe pain imaginable). The VAS score was defined as the mean of the worst daily pain intensity ratings during paroxysms over the preceding 7 days. Additionally, the Self-Rating Anxiety Scale (SAS) and Self-Rating Depression Scale (SDS) were used to evaluate the mental status of all participants^14^. This study was approved by the Ethics Committee of Lanzhou University Second Hospital (approval No. 2024A-1408). In accordance with the Declaration of Helsinki, written consent was obtained from every participant after a detailed explanation of the study.

### Image acquisition

Imaging data were acquired using a 3.0 T MRI scanner (Ingenia CX, Philips Healthcare, the Netherlands) with a 32-channel head/neck coil. All participants were instructed to remain awake, keep their eyes closed and relaxed and avoid active thinking. Noise and head motion were minimized using foam padding and earplugs.

**Figure 1:**
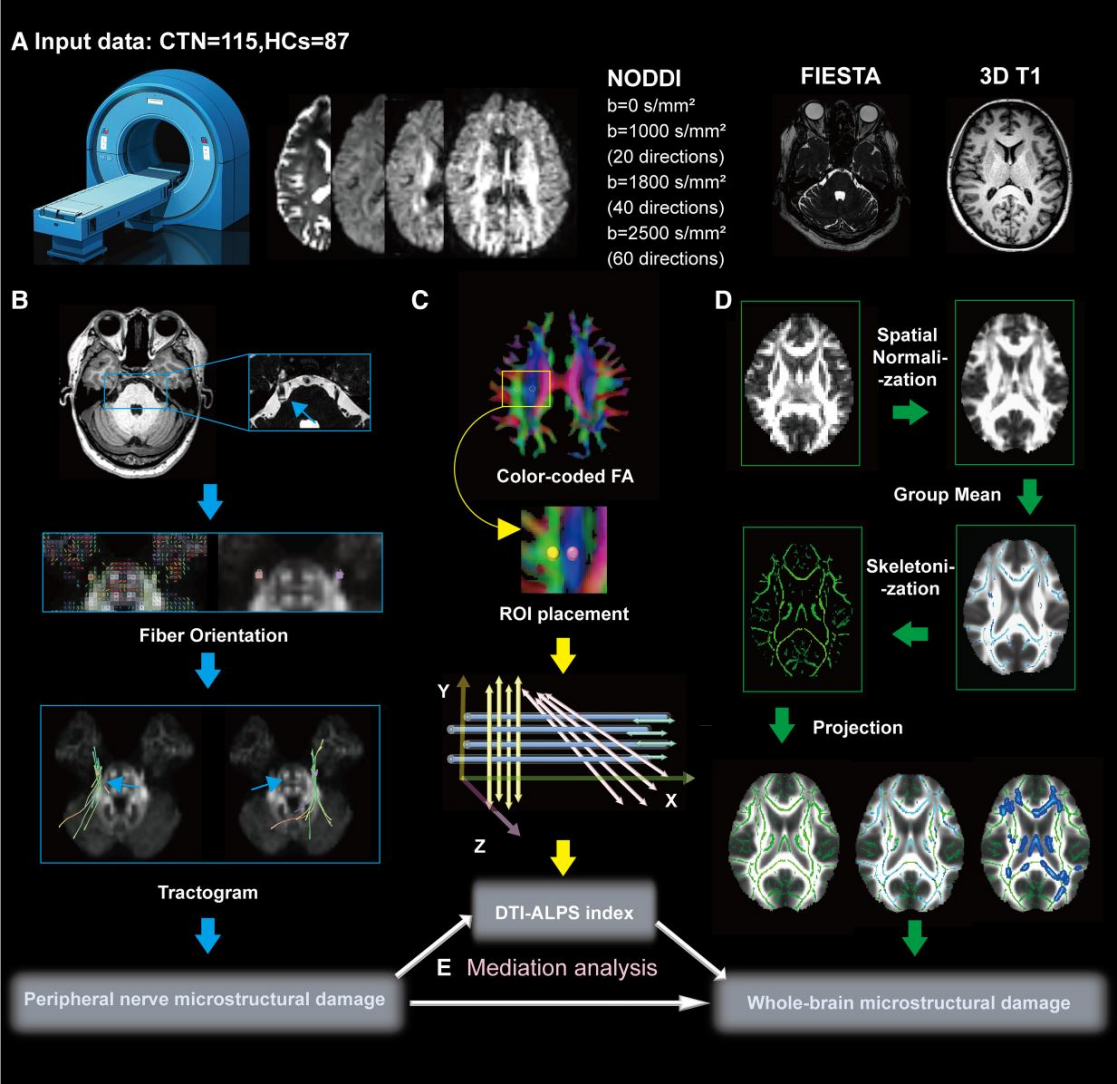
**An overview of the processing pipeline.** (**A**) Data collection. (**B**) Calculating the average diffusion parameters in the NVC. (**C**) Calculating DTI-ALPS index. (**D**) TBSS analysis. (**E**) Mediation analysis. 3DT1, 3-dimensional T1-weighted imaging .

All participants underwent the following scans: (i) multi-shell diffusion MRI (NODDI sequence): repetition time (TR) = 5000 ms; echo time (TE) = 102 ms; spatial resolution = 2 × 2 × 2 mm^3^; diffusion-encoding in 20 directions at b = 1000 s/mm^2^, 40 directions at b = 1800s/mm^2^ and 60 directions at b = 2500 s/mm^2^; one b = 0 s/mm^2^ image with reversed phase-encoding direction; (ii) high-resolution fast imaging employing steady-state acquisition (FIESTA) sequence for NVC visualization: TR = 5.1 ms; TE = 1.9 ms; flip angle = 60°; matrix size = 320 × 288; field of view (FOV) = 20 × 17 cm^2^; slice thickness = 1 mm; no gap; 50 slices; spatial resolution = 0.63 × 0.59 × 1 mm^3^ and (iii) 3D T1-weighted imaging (for anatomical reference and pathology exclusion): TR = 5.9 ms; TE = 3.7 ms; flip angle = 8°; FOV = 256 × 256 mm^2^; isotropic voxel size = 1 × 1 × 1 mm^3^ (Fig. 1.A).

### MRI data processing

All MRI images were independently analysed by two experienced radiologists blinded to the clinical data to ensure objectivity and reliability.

NODDI data pre-processing

The NODDI sequence data were pre-processed and analysed using the FMRIB Software Library (FSL, version 6.0.1, https://www.fmrib.ox.ac.uk/fsl).^15^ First, the acquired images underwent eddy current distortion correction and rigid-body head motion correction to reduce image artefacts and motion-related interference. Subsequently, the Brain Extraction Tool provided by FSL was applied to the *b* = 0 s/mm^2^ images to perform skull stripping, thereby removing the influence of non-brain tissues.^16^ The CUDA Diffusion Modelling Toolbox (cuDIMOT, https://users.fmrib.ox.ac.uk/~moisesf/cudimot/index.html) running on a graphics processing unit (GPU) was employed for NODDI model fitting. Through this model, parameter maps describing the NDI (intracellular volume fraction, reflecting neurite density and integrity), ODI (extracellular volume fraction, reflecting neurite orientation dispersion and complexity) and fraction of isotropic water (FISO; free water content, indicating perineural oedema and inflammatory changes) were generated.^17^ DTI metrics (FA, MD, axial diffusivity (AD), RD) were derived using the FSL Diffusion Toolbox following standard protocols.

Average diffusion parameters at the NVC site

We utilized DSI-Studio software (http://dsi-studio.labsolver.org/) to reconstruct and analyse fibre tracts for all participants^18^. The diffusion images were imported into the DSI-Studio software, a brain mask was applied and generalized q-sampling imaging with a diffusion sampling length ratio of 1.25 was used to reconstruct the model. In the quantitative anisotropy map, the region of interest (ROI) was precisely localized to the NVC site. To ensure consistency, the ROIs in the HCs and on the unaffected side were matched to the location of the ROI on the affected side in CTN patients. The deterministic fibre tracking algorithm was employed to reconstruct the trigeminal nerve tracts, with the following tracking parameters: the quantitative anisotropy threshold was 0.20 ± 0.25 (subject-dependent), the angular threshold was 60°, the step size was 1.2 mm, the smoothness was 0.80, the minimum length was 10 mm and the maximum length was 200 mm. A total of 5000 streamlines were calculated.^19^ Finally, the average diffusion parameters within each ROI were calculated (Fig. 1.B).

Calculation of the DTI-ALPS Index

Diffusion tensor maps were computed from the *b* = 1000 s/mm^2^ shell of the NODDI acquisition, providing directional diffusivities along the x-axis, y-axis and z-axis. The colour-coded FA maps generated from DTI measurements were aligned to the International Consortium for Brain Mapping DTI-81 atlas, and two spherical ROIs (radius = 3 mm) were defined at the level of the unilateral lateral ventricle body: projection fibres (primarily oriented along the z-axis) and association fibres (primarily oriented along the y-axis) (Fig. 1.C). Within the ROIs, diffusivities along the x-axis and y-axis for projection fibres (D_x_proj_, D_y_proj_) and along the x-axis and z-axis for association fibres (D_x_assoc_, D_z_assoc_) were measured. Following the description by Taoka *et al*.^20^, the DTI-ALPS index was calculated by dividing the average of D_x_proj_ and D_x_assoc_ by the average of D_y_proj_ and D_z_assoc_. The DTI-ALPS indices for both hemispheres in CTN patients and HCs were statistically analysed, and the total DTI-ALPS index was defined as the average of the left and right DTI-ALPS indices.

TBSS analysis of left- and right-sided pain groups

Given the unilateral pain characteristics of CTN patients and the potential differences in brain function between the left and right hemispheres, this study compared the left- and right-sided pain groups with HCs separately in the TBSS analysis. The TBSS tool within the FSL software package was used to perform voxel-wise analysis of whole-brain WM measurements.^21^ The nonlinear registration algorithm implemented using FNIRT (FMRIB's Nonlinear Image Registration Tool) aligned the FA images of all participants to the mean FA image template (FMRIB-58) in Montreal Neurological Institute (MNI) space, ensuring spatial consistency.^22^ A mean FA image was generated and thinned to obtain the mean WM skeleton with an FA threshold of 0.3. Each subject’s aligned FA image was then projected onto the mean FA skeleton. This process was repeated for each subject’s MD, AD, RD, NDI, ODI, FISO maps using the individual registration and projection vectors obtained in the FA nonlinear registration and skeletonization^6^ (Fig. 1.D).

### Statistical analysis

Categorical variables were assessed for group differences using the chi-square test (effect size: φ). The normality of continuous variables was evaluated using the Shapiro-Wilk test. Normally distributed continuous variables were expressed as mean ± standard deviation (SD), and paired samples were analysed using the paired *t*-tests (effect size: Cohen’s *d*), while independent samples were compared using the independent samples *t*-tests (effect size: Cohen’s *d*). For non-normally distributed quantitative variables, median and interquartile range (IQR) were used and paired samples were analysed using the Wilcoxon signed-rank tests (effect size: r), while independent samples were compared using the Mann–Whitney *U* tests (effect size: r). These tests were used to compare demographic and clinical variables (age, sex, years of education, SAS, SDS) between CTN patients and HCs.

To address the first research question—Analyses of peripheral microstructural alterations and DTI-ALPS

Intra-patient comparison

Paired *t*-tests were used to compare diffusion metrics and the ipsilateral DTI-ALPS index between the affected and unaffected sides within CTN patients.

Inter-group comparison

A series of one-way analyses of covariance (ANCOVA) were conducted. In each model, the dependent variable was a single diffusion metric or the (ipsilateral/total) DTI-ALPS index. Group (affected side in CTN patients versus anatomically corresponding side in HCs) served as the independent variable. All models included age, sex, years of education and disease duration (for HCs, disease duration was set to 0) as covariates (effect size: η^2^).^23^

Sensitivity analyses

- The primary ANCOVA models were re-run with an additional covariate: the presence of multiple NVCs (binary: yes/no) on the affected nerve in CTN patients (*n* = 19 of 104 patients had multiple contacts).
- To examine the potential influence of incidental NVC in HCs, an ANCOVA within the HC group compared the side with NVC in the ‘incidental NVC’ subgroup (*n* = 11) to the corresponding side in HCs without NVC (*n* = 76), adjusting for age, sex and education.

Exploratory analysis: Influence of offending vessel type

In the subgroup of CTN patients with definitive intraoperative records (*n* = 55), an exploratory ANCOVA was performed. Patients were dichotomized into arterial (*n* = 48) and non-arterial (*n* = 7, combining veno-arterial and venous) compression groups. The model compared diffusion metrics and the DTI-ALPS index between groups, adjusting for age, sex, years of education and disease duration.

To address the second research question—TBSS analyses of whole-brain microstructure

Group comparisons between the TBSS results of CTN and HCs were performed using a general linear model, with age, sex, years of education, disease duration as covariates. To control for multiple comparison errors, threshold-free cluster enhancement and 5000 permutation tests were applied for multiple testing correction in each contrast. Significant voxels were determined by controlling the family-wise error (FWE) rate at a corrected *P_FWE_* < 0.050. To further interpret and visualize the WM regions with significant differences, the Johns Hopkins University (JHU) WM tractography atlas was applied, and the statistically significant results were overlaid on the JHU atlas to intuitively identify and label specific WM fibre tracts.

To address the third research question—Mediation analyses

To test the mediating role of glymphatic function in the relationship between peripheral and widespread WM damage, we performed mediation analyses using Model 4 of the SPSS PROCESS (version 4.1) with 5000 bootstrap iterations. The model examined whether the DTI-ALPS index on the affected side mediated the association between a diffusion parameter at the NVC site and the same diffusion parameter within significant TBSS clusters. All analyses were adjusted for age, sex, years of education and disease duration. A significant indirect mediation effect was concluded if the 95% bias-corrected bootstrap confidence interval (CI) did not include zero.^24^ The mediated effect was calculated as the indirect effect divided by the total effect.^25^

Association with neuropsychological scores

Separate linear regression analyses were conducted with each neuropsychological score (VAS, SAS, SDS) as the dependent variable. Predictors were tested in distinct models and included (i) diffusion metrics at the NVC site, (ii) the ipsilateral and total DTI-ALPS indices and (iii) diffusion metrics within significant TBSS clusters. All models were adjusted for age, sex, years of education and disease duration. Standardized beta coefficients (β) are reported to indicate the direction and strength of associations.

Diagnostic performance analysis

We assessed the diagnostic value of imaging markers using receiver operating characteristic (ROC) analysis within a unified 5-fold cross-validation framework. This approach was applied to calculate the area under the curve (AUC) for peripheral diffusion metrics, the affected side and total DTI-ALPS indices and central diffusion metrics within significant TBSS clusters. To assess combined diagnostic power, multivariable logistic regression models integrating variables along the ‘peripheral–DTI-ALPS index–central’ pathway were also evaluated under the same cross-validation scheme, with all variables Z-score standardized within each training fold. For all cross-validated ROC curves, we report the AUC (95% CI; DeLong method), along with the sensitivity and specificity at the Youden’s index-defined threshold.

Statistical analyses were performed using SPSS (version 27.0) and R (version 4.3.3). For analyses involving multiple comparisons, the false discovery rate (FDR) was controlled using the Benjamini–Hochberg procedure. Effects with a *P_FDR_* value < 0.050 were considered statistically significant.

## Results

### Demographic and clinical characteristics

Among the initially enrolled 115 patients, 5 were excluded due to persistent pain, 2 due to a history of MVD, 1 due to discomfort and inability to complete the MRI examination and 3 due to a cerebellopontine angle lesion. Ultimately, 104 CTN patients were included (53 females, 51 males; age 58.95 ± 9.03 years), among whom 62 had right-sided pain and 42 had left-sided pain. The disease duration ranged from 6 to 360 months, with a median of 32 months. The HCs consisted of 87 volunteers (45 females, 42 males; age 56.57 ± 7.76 years). There were no significant differences in demographic characteristics (including age, sex and education level) between the CTN and HCs (*P* > 0.050), these variables were conservatively included as covariates in all subsequent group-level imaging analyses to control for their potential influence. However, the psychological assessment results of the CTN group showed that their SDS and SAS scores were significantly higher than those of the HCs (*P* < 0.001; Table 1). Additionally, no statistically significant differences were observed in other clinical characteristics and demographic variables between the left- and right-sided pain groups (*P* > 0.050; Table S1).

**Table 1:** Demographic and clinical characteristics of participants

	Patients with CTN (*n* = 104)	HCs (*n* = 87)	φ/Cohen’s *d*/*r*-value	*P*-value
Sex (female/male)	53/51	45/42	0.008	0.916
Age (y), mean ± SD	58.95 ± 9.03	56.57 ± 7.76	0.280	0.055
Education (y), mean ± SD	9.48 ± 5.03	10.22 ± 4.73	0.151	0.301
Duration of disease (m), Median (IQR)	32(16.5, 60)	NA	NA	NA
Attack side(*n*)	Right (62); Left (42)	NA	NA	NA
Follow-up surgery(*n*)	MVD (55); Radiofrequency therapy (6); No surgery/unknown (43)	NA	NA	NA
Medication(*n*)	Carbamazepine (69); Mecobalamin (12); traditional Chinese medicine (11); None (12)			
Score of VAS (score), Median (IQR)	8 (7, 10)	NA	NA	NA
Score of SAS (score), Median (IQR)	46 (34, 58)	34(29, 38)	0.189	**<0.001**
Score of SDS (score), Median (IQR)	47 (35.25, 63.75)	34(28, 39)	0.289	**<0.001**

φ, effect size of chi-square test; Cohen’s *d*, effect size of independent samples *t*-test; r, effect size of Mann–Whitney *U* test.

Bold values indicate statistical significance (*P* < 0.05).

y, years.

### Peripheral diffusion parameters of trigeminal nerve

**Figure 2:**
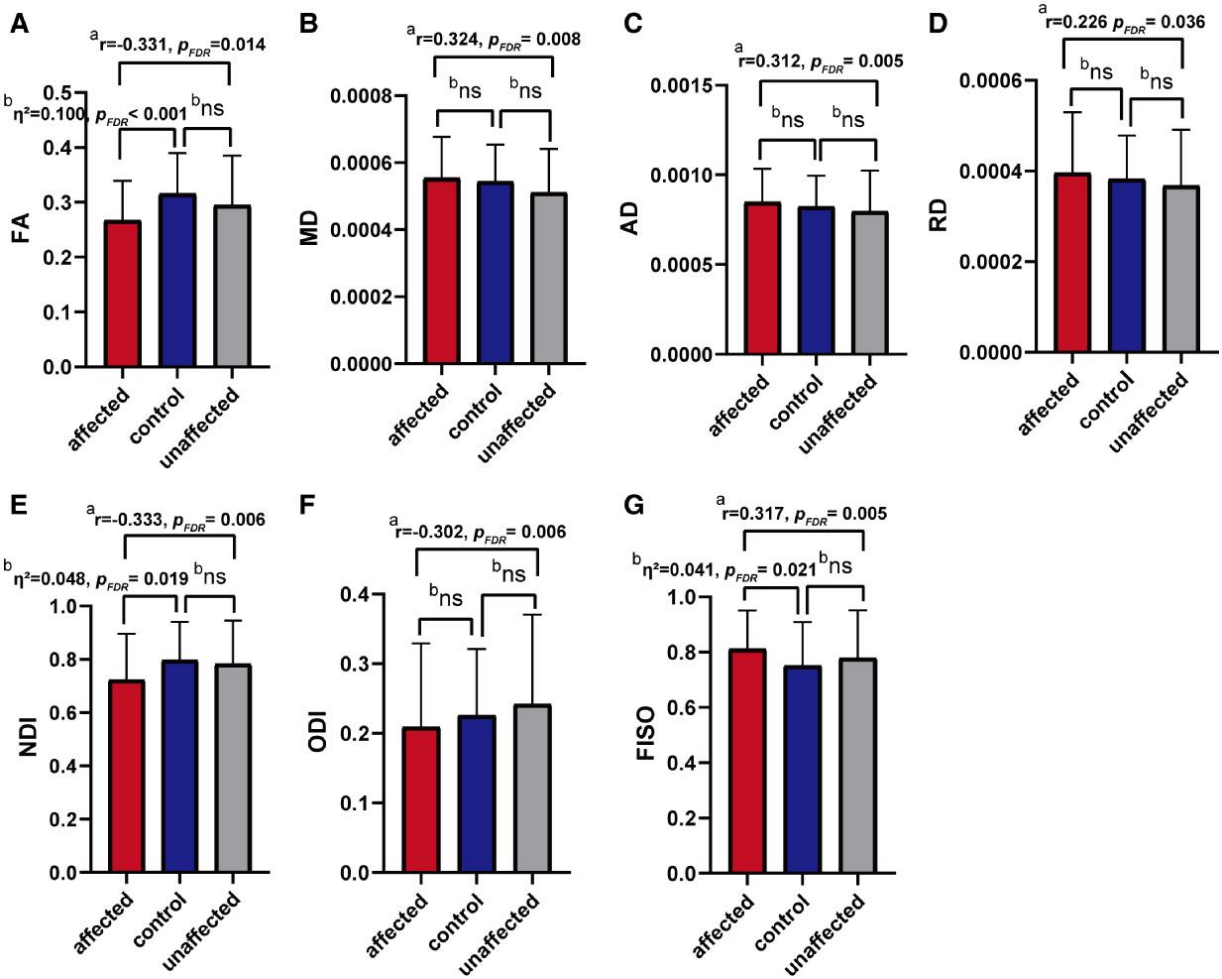
**Group comparisons of peripheral diffusion metrics of the trigeminal nerve.** The bar graphs depict comparisons of DTI/NODDI metrics at the NVC site (affected side) with the unaffected side in CTN patients and with HCs. (**A**) FA. (**B**) MD. (**C**) AD. (**D**) RD. (**E**) NDI. (**F**) ODI. (**G**) FISO. a, paired comparisons between the affected and unaffected sides within CTN patients were assessed using Wilcoxon signed-rank tests (effect size: r). b, group comparisons among the affected side/unaffected side and HCs were performed using analyses of ANCOVA models, with age, sex, years of education and disease duration as covariates (effect size: η^2^). ns, not significant. *P_FDR_*-value, FDR-corrected *P* value. Sample sizes: *n* = 104 for both the affected and unaffected sides in CTN patients; *n* = 87 for HCs.

Compared to the unaffected side, the affected side of CTN patients showed significantly lower FA (*P_FDR_* = 0.014), NDI (*P_FDR_* = 0.006), ODI (*P_FDR_* = 0.006), while MD (*P_FDR_* = 0.008), AD (*P_FDR_* = 0.005), RD (*P_FDR_* = 0.036) and FISO (*P_FDR_* = 0.005) were significantly higher. Compared to the HCs, the affected side of CTN patients exhibited significantly lower FA (*P_FDR_* < 0.001) and NDI (*P_FDR_* = 0.019), while FISO (*P_FDR_* = 0.021) was higher. No significant differences were observed in any diffusion parameters between the unaffected side of CTN patients and the HCs (*P_FDR_* > 0.050) (Fig. 2, Table 2).

**Table 2:** Pairwise comparisons of DTI/NODDI parameters between three groups

Metrics	Affected side versus unaffected side *r*-*value*	*P*-value	*p_FDR_*-value	Affected side versus control η^2^-*value*	*P*-value	*p_FDR_*-value	unaffected side versus control η^2^-*value*	*P*-value	*p_FDR_*-value
DTI metrics									
FA	−0.331	**0.002**	**0.014**	0.100	**< 0.001**	**< 0.001**	0.015	0.082	0.286
MD	0.324	**0.002**	**0.008**	0.002	0.488	0.488	0.019	0.069	0.286
AD	0.312	**0.004**	**0.005**	0.004	0.432	0.488	0.005	0.307	0.394
RD	0.226	**0.036**	**0.036**	0.003	0.450	0.488	0.005	0.338	0.394
NODDI metrics									
NDI	−0.333	**0.003**	**0.006**	0.048	**0.005**	**0.019**	0.001	0.553	0.553
ODI	−0.302	**0.005**	**0.006**	0.005	0.461	0.488	0.006	0.245	0.394
FISO	0.317	**0.004**	**0.005**	0.041	**0.009**	**0.021**	0.007	0.277	0.394

*r*, effect size of Wilcoxon signed-rank test; η^2^, effect size of analyses of covariance; *P_FDR_*-value, false discovery rate -corrected *P* value.

Bold values indicate statistical significance (*P* < 0.05).

Sensitivity analyses, which further adjusted for multiple contacts on the same nerve, yielded similar findings (Table S2). Among HCs, local diffusion parameters did not differ between subgroups with and without incidental NVC (Table S3). In the surgical-confirmed CTN subgroup, no significant differences were found between arterial and non-arterial compression types (Table S4).

Linear regression showed that higher affected side FISO was significantly associated with higher VAS pain scores after multiple comparison correction (*P_FDR_* = 0.013; Table S5).

### Calculation of DTI-ALPS index

**Figure 3:**
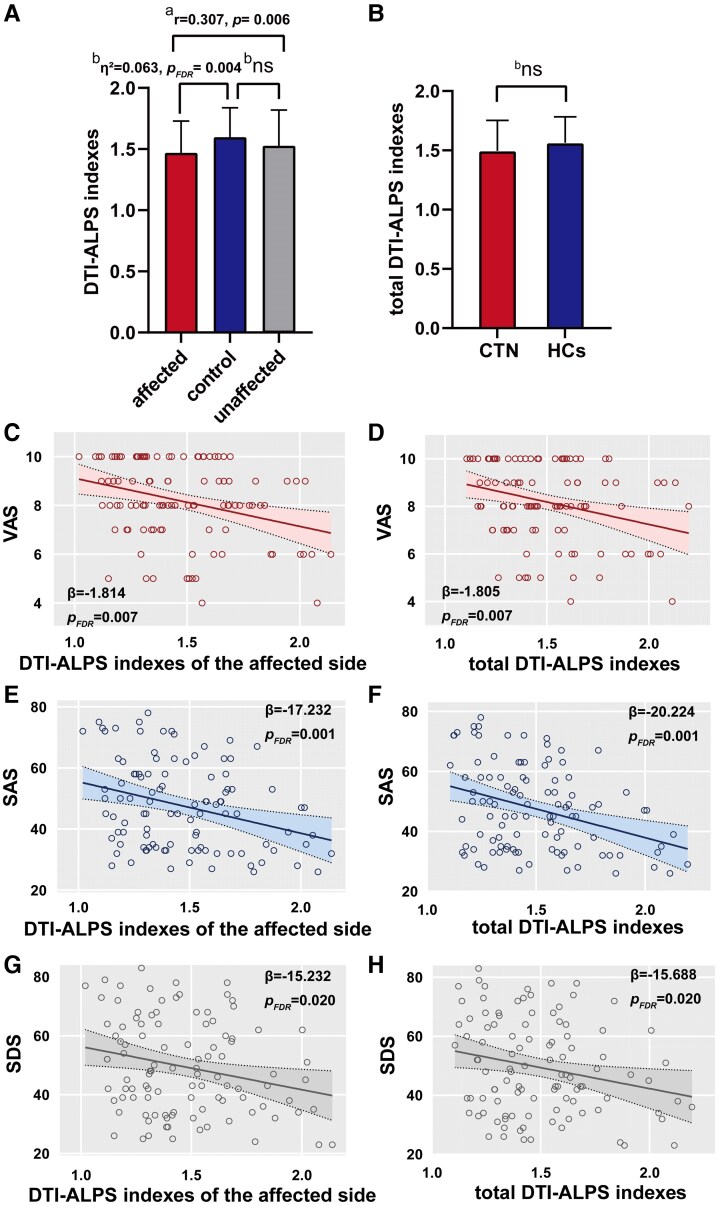
**Group comparisons of DTI-ALPS and correlation analysis.** (**A–B**) Bar graphs showing group comparisons of the affected-side/total DTI-ALPS index. (**A**) Comparison of the unilateral DTI-ALPS index among the affected side, unaffected side of CTN patients and the corresponding side in HCs. (**B**) Comparison of the total DTI-ALPS index between CTN patients and HCs. a, paired comparisons between the affected and unaffected sides within CTN patients were assessed using Wilcoxon signed-rank tests (effect size: r). b, group comparisons among the affected side/unaffected side and HCs were performed using analyses of ANCOVA models, with age, sex, years of education and disease duration as covariates (effect size: η^2^). ns, not significant. *p_FDR_*-value, false discovery rate -corrected *P* value. Sample sizes: *n* = 104 for both the affected and unaffected sides in CTN patients; *n* = 87 for HCs. (**C–H**) Scatter plots showing correlations between the affected-side/total DTI-ALPS index and clinical scales. Each data point in the scatter plots represents the DTI-ALPS index and clinical scale score from one individual CTN patient. (**C–D**) Correlation of the affected-side (**C**) and total (**D**) DTI-ALPS index with pain intensity (VAS). (**E-F**) Correlation of the affected-side (**E**) and total (**F**) DTI-ALPS index with anxiety symptoms (SAS). (**G–H**) Correlation of the affected-side (**G**) and total (**H**) DTI-ALPS index with depressive symptoms (SDS). All six correlation analyses were performed using separate linear regression models, each adjusted for age, sex, years of education and disease duration (effect size: β). *p_FDR_*-value, false discovery rate -corrected *P* value. Sample sizes: *n* = 104 for all linear regression models in CTN patients.

Compared to the unaffected side (*P* = 0.006) and the unilateral side of HCs (*P_FDR_* = 0.004), the DTI-ALPS indexes on the affected side of CTN patients were significantly lower. The DTI-ALPS indexes on the unaffected side of CTN patients showed no significant difference compared to the unilateral side of HCs (*P_FDR_* = 0.109). The total DTI-ALPS indexes of CTN patients also showed no significant difference compared to that of HCs (*P_FDR_* = 0.109) (Fig. 3.A, Table 3).

**Table 3:** Pairwise comparisons of DTI-ALPS index between three groups

Metrics	Affected side versus unaffected side *r*-*value*	*P*-value	*p_FDR_*-value	Affected side versus control η^2^-*value*	*P*-value	*p_FDR_*-value	unaffected side versus control η^2^-*value*	*P*-value	*p_FDR_*-value
Unilateral DTI-ALPS index	0.307	**0.006**	NA	0.063	**0.001**	**0.004**	0.019	0.109	0.109
Total DTI-ALPS index	NA	NA	NA	0.021	0.082	0.109	NA	NA	NA

*r*, effect size of Wilcoxon signed-rank test; η^2^, effect size of analyses of covariance; *p_FDR_*-value, false discovery rate -corrected *P* value; NA, not applicable.

Bold values indicate statistical significance (*P* < 0.05).

Sensitivity analyses, which further adjusted for multiple contacts on the same nerve, yielded similar findings (Table S6). Among HCs, unilateral/total DTI-ALPS indexes did not differ between subgroups with and without incidental NVC (Table S7). In the surgical-confirmed CTN subgroup, no significant differences were found between arterial and non-arterial compression types (Table S8).

Lower affected side DTI-ALPS indexes were negatively correlated with VAS (*P_FDR_* = 0.007), SAS (*P_FDR_* = 0.001) and SDS (*P_FDR_* = 0.020), and lower total DTI-ALPS indexes were significantly negatively correlated with higher VAS (*P_FDR_* = 0.007), SAS (*P_FDR_* = 0.001) and SDS (*P_FDR_* = 0.020) (Fig. 3.B).

### TBSS analyses of whole-brain voxels in left-/right-side pain groups

**Figure 4:**
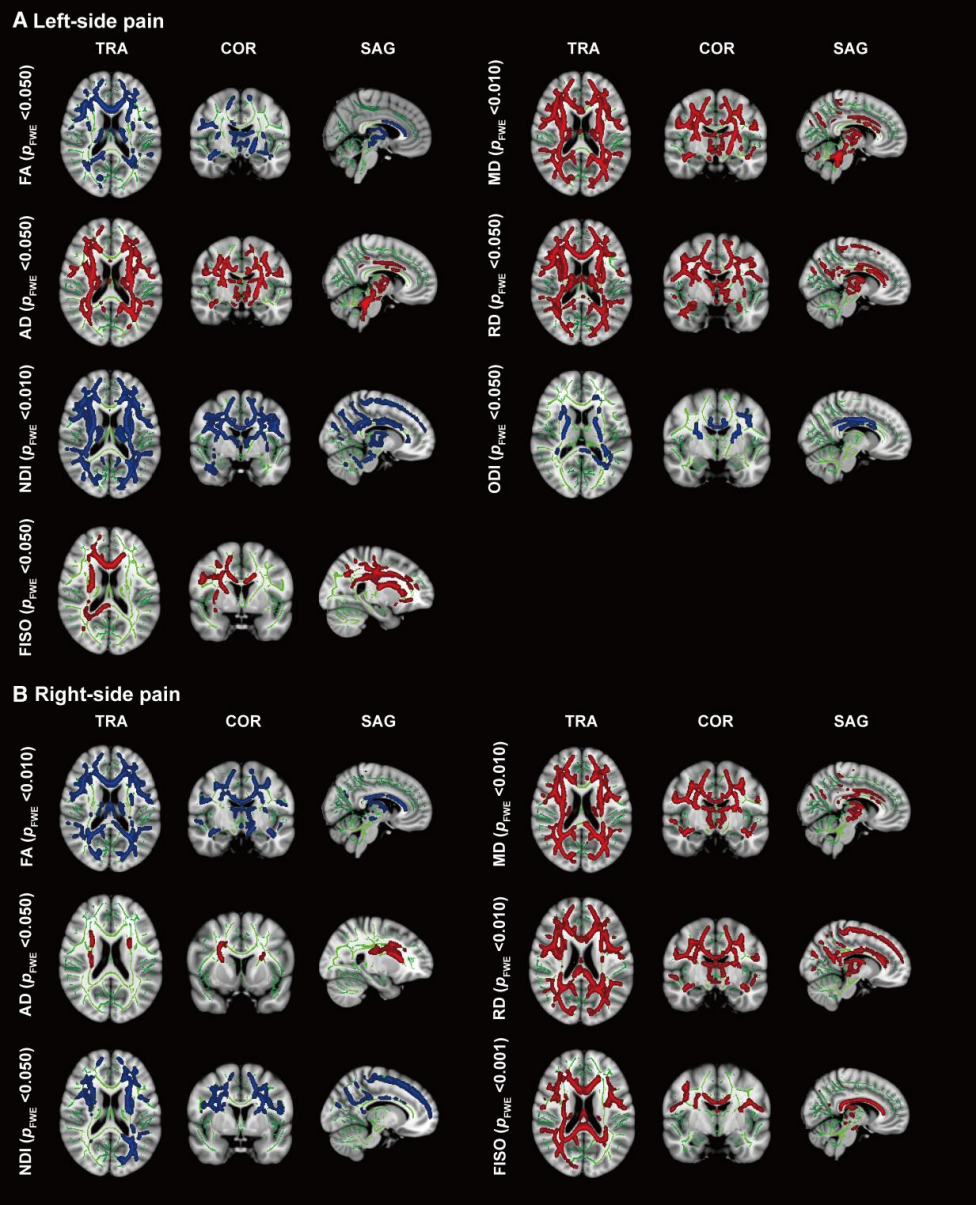
**Visualized TBSS analyses of left/right-side pain in CTN patients.** (**A**) Visualized TBSS analyses of left-side pain in CTN patients based on DTI and NODDI. (**B**) Visualized TBSS analyses of right-side pain in CTN patients based on DTI and NODDI. Red represents the CTN group voxels with significantly higher values than the HCs group. Blue represents the CTN group voxels with significantly lower values than the HCs group. Green denotes the mean FA skeleton. For each pain-side group (**A**) and (**B**), general linear models were conducted at each voxel on the FA skeleton for all diffusion metrics, with age, sex, years of education, disease duration as covariates. Results were corrected for multiple comparisons across space using FWE correction at a threshold of *P* < 0.05. Sample sizes: For (**A**), left-side pain CTN patients: *n* = 62; HCs: *n* = 62. For (**B**), right-side pain CTN patients: *n* = 42; HCs: *n* = 42. SAG, sagittal plane; TRA, transverse plane; WM, white matter.

Compared with HCs, TBSS analyses demonstrated distinct WM microstructural alterations in both left- and right-sided CTN patients (*P_FWE_* < 0.050). In left-sided pain patients, widespread reductions in FA, NDI and ODI, along with higher AD, MD, RD and FISO were observed in bilateral sensorimotor and associative fibres, such as the anterior thalamic radiation (ATR), corticospinal tract (CST), superior longitudinal fasciculus (Fig. 4.A, Table S9). Similarly, right-sided pain patients exhibited extensive WM alterations, characterized by lower FA, NDI and higher MD, AD, RD and FISO values (Fig. 4.B, Table S10).

Linear regression showed that the associations between the significant TBSS tracts of the left-/right-side pain groups and neuropsychological scores did not survive FDR correction (Table S11).

### Results of the mediation analyses

**Figure 5:**
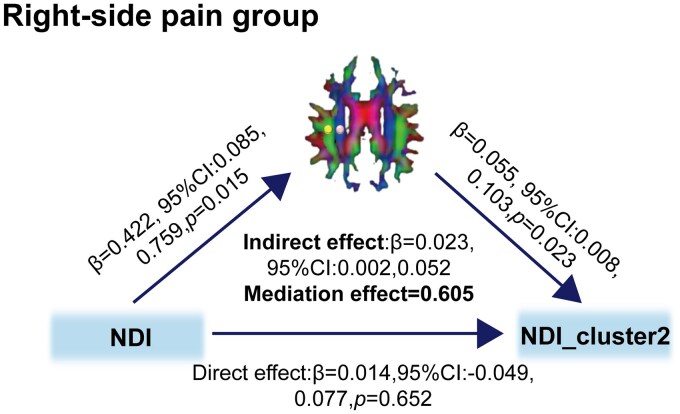
**Results of mediation analyses.** In CTN patients with right-sided pain, the mediating role of the right hemispheric DTI-ALPS indexes in the relationship between the average NDI at the NVC site and the NDI of significant TBSS clusters. Mediation effect = indirect effect/total effect. Sample sizes: *n* = 42 for the right-sided pain group. The experimental unit is individual patient.

The results of all mediation analyses are presented in Table S12. A statistically significant mediation effect was identified in only one pathway: among patients with right-sided pain, the reduction in NDI at the NVC site had a significant indirect effect on NDI reduction within significant TBSS clusters through the right-sided DTI-ALPS index. This indirect pathway accounted for 60.53% of the total effect (Fig. 5).

### Results of the ROC analyses

Among peripheral diffusion parameters at the NVC site, FA exhibited the highest discriminative power, with a cross-validated AUC of 0.685 (95% CI: 0.610–0.760). The affected-side DTI-ALPS index demonstrated modest diagnostic utility (AUC = 0.647, 95% CI: 0.557–0.714). Within the significant clusters identified by TBSS, several metrics showed discriminative power, with cross-validated AUCs exceeding 0.75. The most notable was TBSS_ODI2 (AUC = 0.811, 95% CI: 0.798–0.944) on the left side (Fig. S1, Table S13).

The integrated models demonstrated superior discriminative power compared to individual metrics. On the left side, the ODI-based combination model achieved an outstanding cross-validated AUC of 0.822 (95% CI: 0.734–0.910). On the right side, the FA-based combination model yielded a similarly high performance (AUC = 0.811, 95% CI: 0.755–0.867) (Fig. S2). The corresponding sensitivity and specificity at the optimal cut-off for each combined model are detailed in Table S14.

## Discussion

Our study is the first to combine the NODDI sequence and the DTI-ALPS index to reveal changes in the peripheral and central microstructures of CTN patients, as well as their underlying mechanisms, providing new insights into the pathophysiological mechanisms of CTN. The NODDI sequence, employing a more sophisticated multi-compartment model, further elucidates the complex orientation of neurites. Our findings indicate that at the NVC site, the trigeminal nerve demonstrates lower FA and NDI but higher FISO relative to the unaffected side and HCs. This imaging pattern suggests the presence of axonal injury, impaired nerve fibre integrity and local neuroinflammation. These pathologies collectively constitute the likely substrate for the positive correlation observed between FISO at the NVC site and clinical pain severity (VAS)^26,27^. The neurite damage, plasticity alterations and neuroinflammation identified in our study also provide direct evidence for understanding the pain mechanisms in CTN. Inflammatory mediators, such as bradykinin and prostaglandins, decrease the threshold of transient receptor potential vanilloid 1 and increase Na^+^ channel excitability via individual receptors located on the peripheral side of the primary afferent nerves, thereby leading to peripheral sensitization and pain generation.^28^ Current research has identified that the interleukin 1 receptor type 1/p-p38 MAPK and c-Abl-p38 pathways may provide potential therapeutic benefits for alleviating neuropathic pain^29^. These findings offer valuable insights into future non-invasive therapeutic strategies targeting neuroinflammation for the treatment of CTN.

TBSS results indicated that in patients with both left- and right-sided pain, changes in the diffusion metrics of bilateral WM fibre tracts were observed, suggesting the presence of microscopic damage and neuroinflammation in these fibre tracts. These affected fibre tracts are primarily involved in pain integration, transmission and emotional processing pathways, including ascending nociceptive pathways connecting the thalamus and primary somatosensory cortex, as well as descending pain modulation pathways.^30,31^ Specifically, the ATR and cingulate gyrus (CG) serve as affective-cognitive hubs for pain,^32,33^ while the cingulum hippocampus (CH), inferior fronto-occipital fasciculus and inferior longitudinal fasciculus mediate visual processing and episodic memory related to pain^34^. We found that the correlation between the microstructure abnormalities of WM tracts in CTN and the emotional characteristics validated the interplay between pain and emotion across different brain regions. The emotional pathway is centred on the limbic system, and facilitates interhemispheric communication, cortical-subcortical integration and prefrontal-limbic connectivity through association fibres (such as the uncinate fasciculus, CG) and commissural fibres (such as corpus callosum).^35^ These WM tract impairments associated with emotional memory also play a critical role in central sensitization of pain, which is an adaptive change in the CNS that amplifies and propagates nociceptive signals.^36^ The CST acts as a descending pathway, transmitting signals from the cerebral cortex to the brainstem to inhibit pain transmission.^37^ In chronic pain, CST impairment reduces pain inhibition at the trigeminal nucleus, leading to increased neuronal excitability in the spinal cord and brainstem, which may further contribute to central sensitization.^38^ The observed correlations between the microstructure changes of central WM and pain intensity in our study further substantiate these findings.

Taoka *et al*.^39^ evaluated the reproducibility of the DTI-ALPS method across different scanners and found that the DTI-ALPS index is robust under consistent imaging protocols. In this study, the affected side DTI-ALPS indexes were lower in CTN patients compared to HCs, indicating impaired glymphatic function in CTN patients. The mediation analysis in this study revealed that the DTI-ALPS index mediates the relationship between peripheral neural injury and whole-brain microstructural changes. Local axonal injury in the trigeminal nerve may release damage-associated molecular patterns, activating microglia and inducing the release of pro-inflammatory factors such as interleukin-1β and tumour necrosis factor-α, leading to aberrant signal transduction and persistent facial pain.^40^ These substances are released into the extracellular space and flow within the GS, causing neuronal degeneration as well as hypertrophy and activation of astrocytes.^41^ This further results in interstitial fluid stagnation, deposition of metabolic waste throughout the brain, and increased time required for the clearance of toxic solutes and waste proteins, exacerbating GS dysfunction and initiating a vicious cycle.^42^ Additionally, the GS is crucial for the removal of reactive oxygen species (ROS). Damage to the GS leads to the accumulation of pro-inflammatory cytokines and ROS. Due to the brain’s high oxygen consumption, lipid metabolism and poor antioxidant capacity, it is highly susceptible to ROS damage.^43^ This ‘peripheral-DTI-ALPS index-central’ pathway provides a plausible mechanistic bridge connecting the focal aetiology of CTN to its widespread central signatures and chronicity. This model, while speculative, offers several novel clinical implications. First, the DTI-ALPS index emerges as a potential imaging biomarker for glymphatic dysfunction in neuropathic pain. Second, it suggests new therapeutic targets. Strategies aimed at enhancing glymphatic function (such as specific pharmacological agents, or non-invasive neuromodulation) could be tested as adjunctive therapies for CTN, particularly in patients with reduced DTI-ALPS indices. Third, it underscores the importance of laterality-specific assessment, as our data show distinct central remodelling patterns for left- versus right-sided pain, which may inform more tailored neuromodulation approaches.^44^ Future longitudinal studies are essential to validate the temporal sequence of this pathway and to determine if modulating glymphatic function can alter the progression of central changes or improve clinical outcomes.

Our diagnostic performance analysis indicated that while single imaging parameters showed modest discriminative utility, integrated models combining features from the peripheral, glymphatic and central levels achieved superior performance. Therefore, future diagnostic models may need to integrate multi-dimensional imaging features, clinical manifestations and even genomic data not only to enhance diagnostic accuracy but also, more importantly, to distinguish underlying pathological subtypes and inform personalized treatment selection. The AUC data provided in this study only lay a preliminary foundation for constructing such multi-parameter models.

Several limitations of this study should be considered. First, the observed statistical associations and mediation effects in our cross-sectional study can only suggest a plausible sequence of events. Longitudinal studies are required to establish the temporal dynamics of these alterations. Second, the DTI-ALPS index, while a practical and non-invasive proxy, primarily assesses glymphatic activity adjacent to the lateral ventricles and may not fully represent the function of the entire system. Third, inflammatory markers were not directly measured. In the future, this pathway can be verified in combination with CSF or blood biomarkers. Fourth, clinical pain assessment relied on VAS scales, which may not adequately capture the paroxysmal nature of CTN episodes. Future studies incorporating prospective digital pain diaries would provide more dynamic and valid metrics. Fifth, the exploratory analysis comparing arterial and non-arterial compression types was limited by a small sample size in the non-arterial group, which may have obscured potential differences. Future studies with larger cohorts are warranted to specifically address the heterogeneity of NVC. Sixth, we focused on patients with purely paroxysmal CTN to ensure cohort homogeneity. Patients with concomitant persistent pain might exhibit more pronounced central sensitization and glymphatic impairment. Direct comparisons between these clinical phenotypes in future work are warranted. Finally, while our sample size was sufficient for the primary univariate and mediation analyses, it is inadequate for developing and validating a robust multi-parameter diagnostic model that integrates peripheral, glymphatic and central markers. Future research with larger cohorts, potentially incorporating fluid biomarkers or genomic data, is needed to build and test such integrative models.

## Conclusions

Our findings point to GS dysfunction as a plausible mechanism connecting peripheral and central pathology in CTN. While preliminary, these findings highlight the GS as a candidate focus for further mechanistic investigation and may inform future approaches to diagnosis and treatment.

## Supplementary Material

fcag191_Supplementary_Data

## Data Availability

Raw data inquiries are available from the corresponding author upon reasonable request.
