# Supplementary material for: Glymphatic system bridges peripheral and central nervous system changes in classic trigeminal neuralgia
Source: Brain Commun. 2026 Jun 3;8(3):fcag191. doi: 10.1093/braincomms/fcag191 (PMC13247850; doi:10.1093/braincomms/fcag191)
Supplement: fcag191_Supplementary_Data [file fcag191_supplementary_data.docx]

**Supplementary Materials**

**Figure S1.** The ROC curves of individual imaging metrics using 5-fold cross-validation. ------------------------------------------------------------------------------------------------page 2

**Figure S2.** The ROC curves of multivariable diagnostic models using 5-fold cross-validation. ----------------------------------------------------------------------------------page 4

**Table S1.** Demographic and clinical characteristics of left- and right-sided CTN patients. -------------------------------------------------------------------------------------page 5

**Table S2.** Sensitivity analysis of diffusion parameters at the neurovascular compression site, with additional adjustment for the presence of multiple contacts. ------------page 6

**Table S3.** Comparison of local diffusion parameters in HCs between subgroups with and without incidental neurovascular compression. ----------------------------------page 8

**Table S4.** Exploratory analysis of diffusion parameters by offending vessel type (arterial vs. non-arterial) in the surgically confirmed patient subgroup. -----------page 9

**Table S5.** Linear regression analysis assessing the association between diffusion parameters at the neurovascular compression site and clinical scores. ------------page 10

**Table S6.** Sensitivity analyses of the DTI-ALPS index, with additional adjustment for the presence of multiple neurovascular contacts. -------------------------------------page 11

**Table S7.** Comparison of the DTI-ALPS index in HCs between subgroups with and without incidental neurovascular compression. --------------------------------------page 12

**Table S8.** Exploratory analysis of the DTI-ALPS index by offending vessel type (arterial vs. non-arterial) in the surgically confirmed patient subgroup. ----------page 13

**Table S9.** TBSS analysis of CTN patients with left-side pain based on DTI and NODDI. ----------------------------------------------------------------------------------------------page 14

**Table S10.** TBSS analysis of CTN patients with right-side pain based on DTI and NODDI. -----------------------------------------------------------------------------------page 16

**Table S11.** Associations between altered white matter tracts (identified by TBSS) and neuropsychological scores in left- and right-sided pain patient groups. -----------page 18

**Table S12.** Results of mediation analyses. -------------------------------------------page 21

**Table S13.** Diagnostic performance of individual imaging metrics for discriminating CTN patients from HCs. -----------------------------------------------------------------page 24

**Table S14.** Diagnostic performance of combined multivariable models for discriminating CTN patients from HCs. -----------------------------------------------page 27


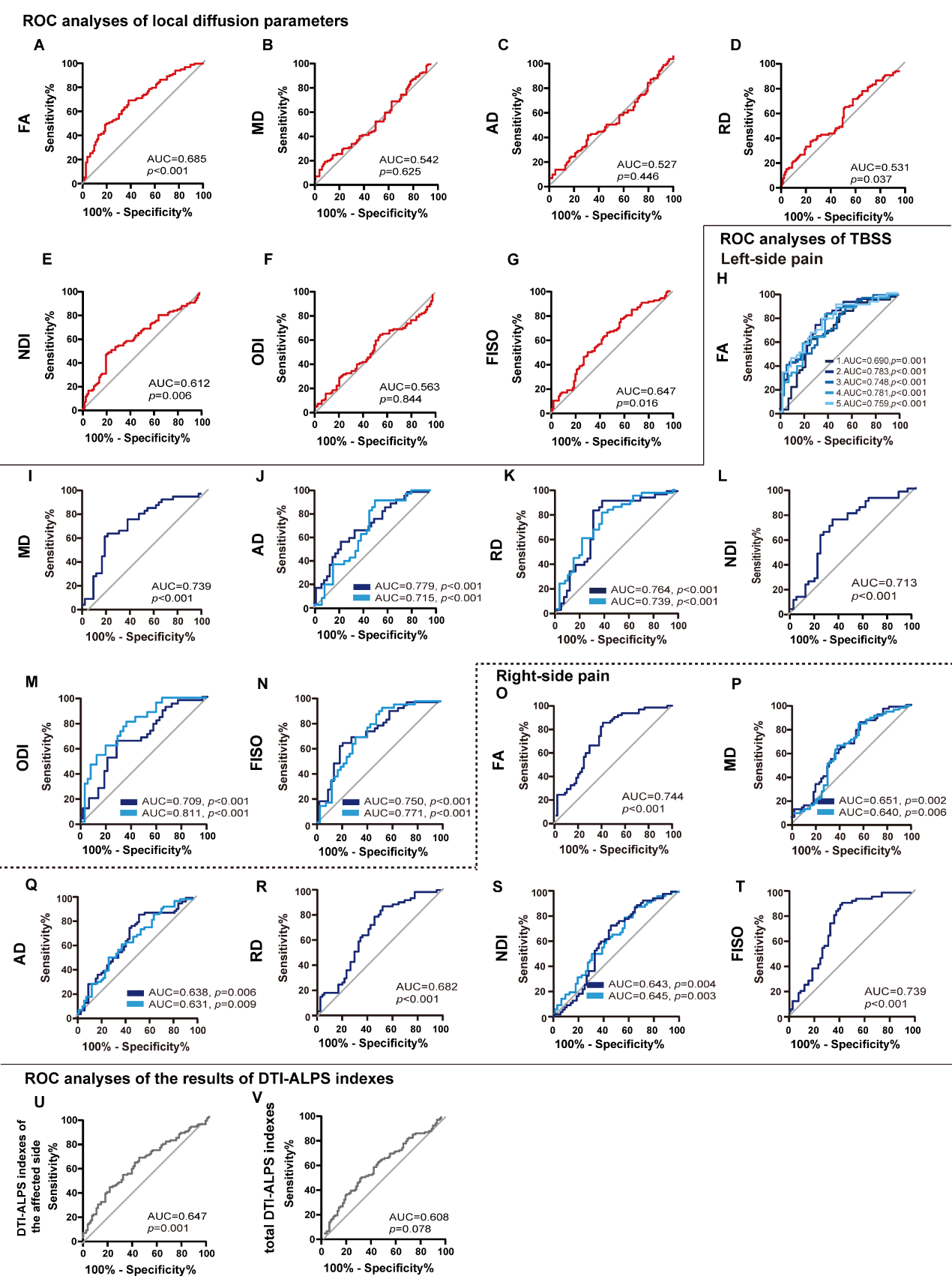


**Figure S1.** **The ROC curves of individual imaging metrics using 5-fold cross-validation.**

(A-G) ROC analyses of diffusion parameters at the NVC site: (A) FA; (B) MD; (C) AD; (D) RD; (E) NDI; (F) ODI; (G) FISO.
(H-T) ROC analyses of the results of TBSS. (H-N): Left-side pain group: (H) FA; (I) MD; (J) AD; (K) RD; (L) NDI; (M) ODI; (N) FISO. (O-T): Right-side pain group: (O) FA; (P) MD; (Q) AD; (R) RD; (S) NDI; (T) FISO.

(U-V) ROC analyses of the DTI-ALPS indexes: (U) DTI-ALPS indexes of the affected side; (V) total DTI-ALPS indexes.
The diagnostic performance of each parameter, quantified by the area under the ROC curve (AUC), was evaluated using 5-fold cross-validation.
Sample sizes: For (A-G), CTN patients: n = 104; HCs: n = 87. For (H-N), left-side pain CTN patients: n = 62; HCs: n = 62. For (O-T), right-side pain CTN patients: n = 42; HCs: n = 42. For (U-V), CTN patients: n = 104; HCs: n = 87.

AD, axial diffusivity; AUC, area under the curve; CTN, classic trigeminal neuralgia; DTI-ALPS, diffusion tensor imaging along the perivascular space; FA, fractional anisotropy; FISO, fraction of isotropic diffusion; HCs, healthy controls; MD, mean diffusivity; NDI, neurite density index; NVC, neurovascular contact; ODI, orientation dispersion index; RD, radial diffusivity; ROC, receiver operating characteristic; TBSS, Tract-based spatial statistics.

**
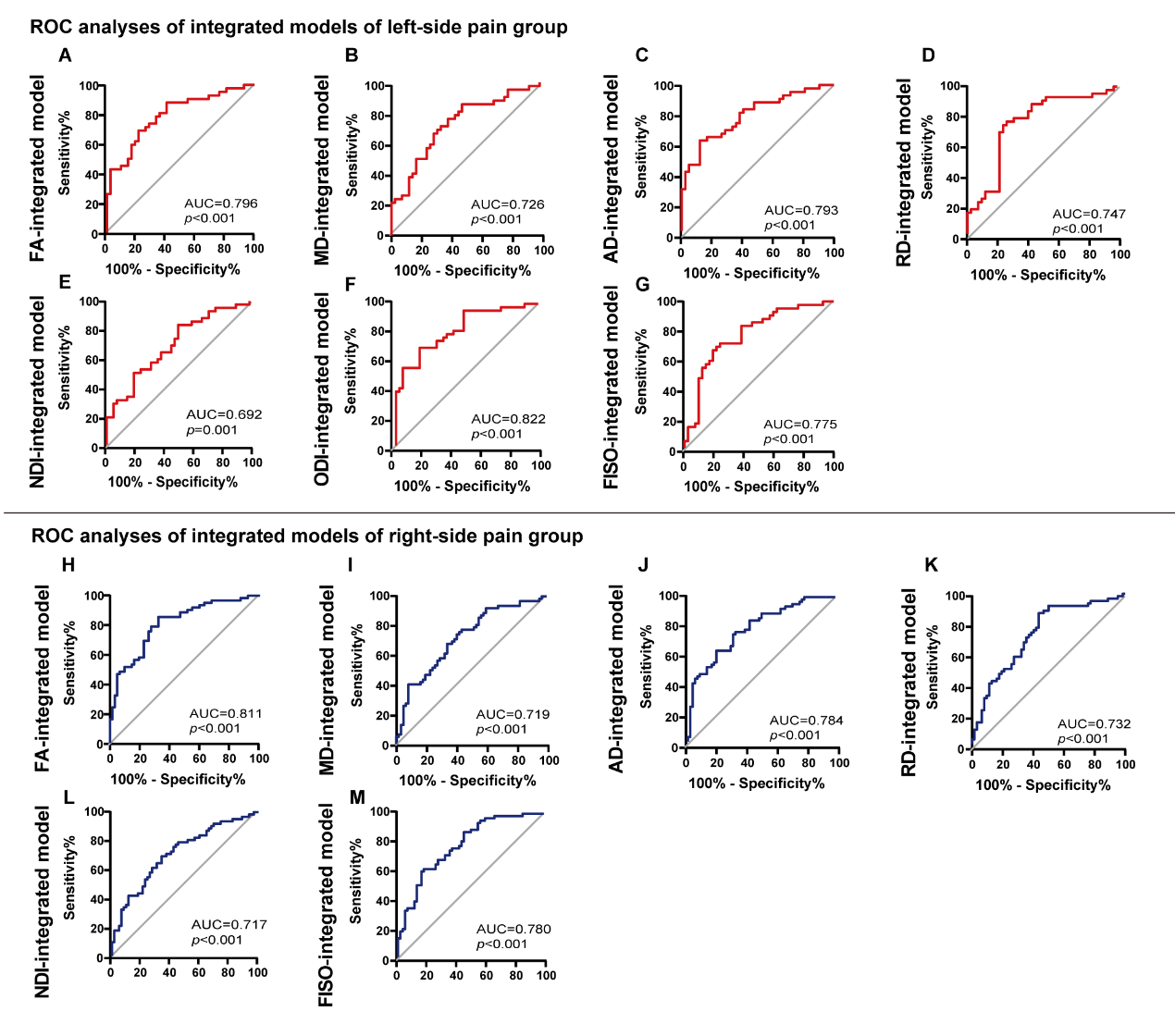
**

**Figure S2.** **The ROC curves of multivariable diagnostic models using 5-fold cross-validation.**

(A-G) ROC analyses of integrated models for the left-side pain group. (A) FA; (B) MD; (C) AD; (D) RD; (E) NDI; (F) ODI; (G) FISO.

(H-M) ROC analyses of integrated models for the right-side pain group. (H) FA; (I) MD; (J) AD; (K) RD; (L) NDI; (M) FISO.

To assess the combined diagnostic power, multivariable logistic regression models integrating variables along the ‘peripheral–DTI‑ALPS index–central’ pathway were constructed. All model variables were Z‑score standardized within each training fold of the 5-fold cross-validation procedure to evaluate generalizable performance. The diagnostic performance of each model, quantified by the area under the ROC curve (AUC), is reported.

Sample sizes: For panels (A-G), left-side pain CTN patients: n = 42; HCs: n = 42. For panels (H-M), right-side pain CTN patients: n = 62; HCs: n = 62.

AD, axial diffusivity; AUC, area under the curve; CTN, classic trigeminal neuralgia; DTI-ALPS, diffusion tensor imaging along the perivascular space; FA, fractional anisotropy; FISO, fraction of isotropic diffusion; HCs, healthy controls; MD, mean diffusivity; NDI, neurite density index; ODI, orientation dispersion index; RD, radial diffusivity; ROC, receiver operating characteristic.

**Table S1. Demographic and clinical characteristics of left- and right-sided CTN patients.**

| **Left- and right-side pain group** | **Left**  **(*n*=42)** | **Right**  **(*n*=62)** | **φ/Cohen‘s d/r- value** | ***p*-value** |
| --- | --- | --- | --- | --- |
| Sex (female/male) | 21/21 | 32/30 | 0.016 | 0.872 |
| Age(y),mean±SD | 58.88 ± 8.89 | 59.00 ± 9.20 | 0.121 | 0.904 |
| Education(y),mean±SD | 10.10 ± 4.65 | 9.06 ± 5.27 | 0.056 | 0.660 |
| Duration of disease(m), Median(IQR) | 32(16, 48) | 32(17.5, 60.5) | 0.048 | 0.625 |
| Score of VAS(score), Median(IQR) | 8(6, 9) | 8(8, 10) | 0.199 | 0.077 |
| Score of SAS(score), Median(IQR) | 45(33, 54.25) | 48(34, 62) | 0.144 | 0. 214 |
| Score of SDS(score), Median(IQR) | 43(33.75, 56) | 48(38, 65.25) | 0.188 | 0.102 |

φ, effect size of chi-square test; Cohen‘s d, effect size of independent samples t-test; r, effect size of Mann-Whitney U test.

CTN, classic trigeminal neuralgia; IQR, interquartile range; n, number; SAS, self-rating anxiety scale; SD, standard deviation; SDS, self-rating depression scale; VAS, visual analogue scale; y, years.

**Table S2. Sensitivity analyses of diffusion parameters at the neurovascular compression site, with additional adjustment for the presence of multiple contacts.**

| **Metrics** | **Predictors** | **F-value** | ***p*-value** |
| --- | --- | --- | --- |
| FA | group | 15.307 | **<0.001** |
| FA | age | 0.360 | 0.549 |
| FA | gender | 4.992 | 0.027 |
| FA | education | 2.031 | 0.156 |
| FA | Duration | 0.943 | 0.333 |
| FA | multiNVC | 1.297 | 0.256 |
| MD | group | 1.149 | 0.285 |
| MD | age | 2.496 | 0.116 |
| MD | gender | 0.151 | 0.698 |
| MD | education | 3.082 | 0.081 |
| MD | Duration | 1.993 | 0.160 |
| MD | multiNVC | 0.314 | 0.576 |
| AD | group | 1.803 | 0.181 |
| AD | age | 0.053 | 0.818 |
| AD | gender | 1.333 | 0.250 |
| AD | education | 4.270 | 0.040 |
| AD | Duration | 1.237 | 0.268 |
| AD | multiNVC | 0.460 | 0.499 |
| RD | group | 1.151 | 0.285 |
| RD | age | 1.193 | 0.276 |
| RD | gender | 0.357 | 0.551 |
| RD | education | 4.777 | 0.030 |
| RD | Duration | 2.405 | 0.123 |
| RD | multiNVC | 0.978 | 0.324 |
| NDI | group | 4.755 | **0.030** |
| NDI | age | 2.823 | 0.095 |
| NDI | gender | 0.000 | 0.991 |
| NDI | education | 0.042 | 0.838 |
| NDI | Duration | 0.234 | 0.629 |
| NDI | multiNVC | 1.909 | 0.169 |
| ODI | group | 0.018 | 0.893 |
| ODI | age | 0.897 | 0.345 |
| ODI | gender | 0.583 | 0.446 |
| ODI | education | 0.907 | 0.342 |
| ODI | Duration | 1.466 | 0.228 |
| ODI | multiNVC | 0.085 | 0.771 |
| FISO | group | 4.904 | **0.028** |
| FISO | age | 1.411 | 0.236 |
| FISO | gender | 0.987 | 0.322 |
| FISO | education | 0.032 | 0.859 |
| FISO | Duration | 0.003 | 0.958 |
| FISO | multiNVC | 0.005 | 0.943 |

AD, axial diffusivity; FA, fractional anisotropy; FISO, fraction of isotropic diffusion; MD, mean diffusivity; multiNVC, multiple neurovascular contacts; NDI, neurite density index; ODI, orientation dispersion index; RD, radial diffusivity.

**Table S3. Comparison of local diffusion parameters in HCs between subgroups with and without incidental neurovascular compression.**

| **Metrics** | **F-value** | **η²-*value*** | ***p*-value** | ***p_FDR_*-value** |
| --- | --- | --- | --- | --- |
| FA | 1.236 | 0.018 | 0.269 | 0.529 |
| MD | 0.193 | 0.001 | 0.661 | 0.895 |
| AD | 1.222 | 0.014 | 0.272 | 0.529 |
| RD | 0.017 | 0.001 | 0.895 | 0.895 |
| NDI | 2.847 | 0.035 | 0.095 | 0.529 |
| ODI | 0.056 | 0.002 | 0.814 | 0.895 |
| FISO | 1.077 | 0.012 | 0.302 | 0.529 |

η², effect size of analyses of covariance; *p_FDR_*-value, false discovery rate -corrected *p* value.

AD, axial diffusivity; FA, fractional anisotropy; FISO, fraction of isotropic diffusion; HCs, healthy controls; MD, mean diffusivity; NDI, neurite density index; ODI, orientation dispersion index; RD, radial diffusivity.

**Table S4. Exploratory analyses of diffusion parameters by offending vessel type (arterial vs. non-arterial) in the surgically confirmed patient subgroup.**

| **Metrics** | **F-value** | **η²-*value*** | ***p*-value** | ***p_FDR_*-value** |
| --- | --- | --- | --- | --- |
| FA | 0.264 | 0.002 | 0.610 | 0.711 |
| MD | 0.618 | 0.008 | 0.436 | 0.711 |
| AD | 0.540 | 0.014 | 0.466 | 0.711 |
| RD | 0.297 | 0.005 | 0.588 | 0.711 |
| NDI | 3.268 | 0.033 | 0.077 | 0.537 |
| ODI | 0.431 | 0.006 | 0.514 | 0.711 |
| FISO | 0.024 | 0.002 | 0.878 | 0.878 |

η², effect size of analyses of covariance; *p_FDR_*-value, false discovery rate -corrected *p* value.

AD, axial diffusivity; FA, fractional anisotropy; FISO, fraction of isotropic diffusion; MD, mean diffusivity; NDI, neurite density index; ODI, orientation dispersion index; RD, radial diffusivity.

**Table S5. Linear regression analyses assessing the association between diffusion parameters at the neurovascular compression site and clinical scores.**

| **Metrics** | **β** | **se** | **t-value** | ***p*-value** | ***p_FDR_*-value** |
| --- | --- | --- | --- | --- | --- |
| **Correlation with VAS** | | |  |  |  |
| FA | -2.847 | 2.294 | -1.241 | 0.218 | 0.761 |
| MD | 789.424 | 1367.764 | 0.577 | 0.565 | 0.806 |
| AD | -484.827 | 914.934 | -0.530 | 0.597 | 0.806 |
| RD | 774.322 | 1233.843 | 0.628 | 0.532 | 0.806 |
| NDI | -0.383 | 0.959 | -0.399 | 0.691 | 0.806 |
| ODI | -0.190 | 1.364 | -0.139 | 0.890 | 0.890 |
| FISO | 3.619 | 1.128 | 3.209 | 0.002 | **0.013** |
| **Correlation with SAS** | | |  |  |  |
| FA | -29.137 | 19.996 | -1.457 | 0.148 | 0.259 |
| MD | 2447.568 | 11974.540 | 0.204 | 0.838 | 0.859 |
| AD | 12665.160 | 7906.830 | 1.602 | 0.112 | 0.259 |
| RD | -1920.190 | 10805.977 | -0.178 | 0.859 | 0.859 |
| NDI | 6.533 | 8.360 | 0.781 | 0.436 | 0.611 |
| ODI | -26.263 | 11.623 | -2.259 | 0.026 | 0.091 |
| FISO | 26.154 | 10.022 | 2.610 | 0.010 | 0.073 |
| **Correlation with SDS** | | | |  |  |
| FA | -41.185 | 22.414 | -1.837 | 0.069 | 0.242 |
| MD | -5838.748 | 13469.915 | -0.433 | 0.666 | 0.755 |
| AD | 2828.880 | 9057.767 | 0.312 | 0.755 | 0.755 |
| RD | -6725.899 | 12138.736 | -0.554 | 0.581 | 0.755 |
| NDI | 15.379 | 9.374 | 1.641 | 0.104 | 0.243 |
| ODI | -18.014 | 13.410 | -1.343 | 0.182 | 0.319 |
| FISO | 27.295 | 11.330 | 2.409 | 0.018 | 0.125 |

β: Standardized beta coefficient of separate linear regression analysis; *p_FDR_*-value, false discovery rate -corrected *p* value.

AD, axial diffusivity; CTN, classic trigeminal neuralgia; FA, fractional anisotropy; FISO, fraction of isotropic diffusion; MD, mean diffusivity; NDI, neurite density index; ODI, orientation dispersion index; RD, radial diffusivity; SAS, self-rating anxiety scale; SDS, self-rating depression scale; VAS, visual analogue scale.

**Table S6. Sensitivity analyses of the DTI-ALPS index, with additional adjustment for the presence of multiple neurovascular contacts.**

| **Metrics** | **Predictors** | **F-value** | ***p*-value** |
| --- | --- | --- | --- |
| DTI-ALPS indexes of the affected side | group | 4.441 | **0.036** |
| DTI-ALPS indexes of the affected side | age | 1.570 | 0.212 |
| DTI-ALPS indexes of the affected side | gender | 0.591 | 0.443 |
| DTI-ALPS indexes of the affected side | education | 0.377 | 0.540 |
| DTI-ALPS indexes of the affected side | Duration | 0.603 | 0.438 |
| DTI-ALPS indexes of the affected side | multiNVC | 0.755 | 0.386 |
| total DTI-ALPS indexes | group | 0.720 | 0.397 |
| total DTI-ALPS indexes | age | 2.898 | 0.090 |
| total DTI-ALPS indexes | gender | 1.071 | 0.302 |
| total DTI-ALPS indexes | education | 0.002 | 0.961 |
| total DTI-ALPS indexes | Duration | 0.341 | 0.560 |
| total DTI-ALPS indexes | multiNVC | 1.498 | 0.222 |
| DTI-ALPS indexes of the unaffected side | group | 0.707 | 0.401 |
| DTI-ALPS indexes of the unaffected side | age | 3.937 | 0.049 |
| DTI-ALPS indexes of the unaffected side | gender | 2.279 | 0.133 |
| DTI-ALPS indexes of the unaffected side | education | 0.054 | 0.817 |
| DTI-ALPS indexes of the unaffected side | Duration | 0.068 | 0.794 |
| DTI-ALPS indexes of the unaffected side | multiNVC | 1.886 | 0.171 |

DTI-ALPS, diffusion tensor imaging along the perivascular space; multiNVC, multiple neurovascular contacts.

**Table S7. Comparison of the DTI-ALPS index in HCs between subgroups with and without incidental neurovascular compression.**

| **Metrics** | **F-value** | **η²-value** | ***p*-value** | ***p_FDR_*-value** |
| --- | --- | --- | --- | --- |
| DTI-ALPS indexes of the affected side | 0.886 | 0.011 | 0.349 | 0.487 |
| total DTI-ALPS indexes | 0.487 | 0.006 | 0.487 | 0.487 |
| DTI-ALPS indexes of the unaffected side | 0.886 | 0.011 | 0.349 | 0.487 |

η², effect size of analyses of covariance; *p_FDR_*-value, false discovery rate -corrected *p* value.

DTI-ALPS, diffusion tensor imaging along the perivascular space; HCs, healthy controls.

**Table S8. Exploratory analyses of the DTI-ALPS index by offending vessel type (arterial vs. non-arterial) in the surgically confirmed patient subgroup.**

| **Metrics** | **F-value** | **η²*-value*** | ***p*-value** | ***p_FDR_*-value** |
| --- | --- | --- | --- | --- |
| DTI-ALPS indexes of the affected side | 0.296 | 0.011 | 0.589 | 0.589 |
| total DTI-ALPS indexes | 0.408 | 0.001 | 0.526 | 0.589 |
| DTI-ALPS indexes of the unaffected side | 2.481 | 0.022 | 0.122 | 0.365 |

η², effect size of analyses of covariance; *p_FDR_*-value, false discovery rate -corrected *p* value.

DTI-ALPS, diffusion tensor imaging along the perivascular space.

**Table S9. TBSS analysis of CTN patients with left-side pain based on DTI and NODDI.**

| Metrics | Cluster index | Number of voxels | signal peaks (x, y, z) | *p*-value^#^ | White matter tracts |
| --- | --- | --- | --- | --- | --- |
| FA | 1 | 67 | (-24, -68, 28) | 0.047 | ATR_ L; CH_L; F_major; IFOF_L; ILF_L; |
|  | 2 | 187 | (15, -33, 55) | 0.045 | ATR_ R; CST_R; CG_R; SLF_R |
|  | 3 | 312 | (-29, -22, 37) | 0.042 | ATR_ L; CST_L; ILF_L; SLF_L; SLF_ (temporal part) L |
|  | 4 | 7137 | (-28, -29, -5) | 0.028 | ATR_ L; ATR_ R; CST_L; CG_L; CH_L; F_major; F_minor; IFOF_L; ILF_L; SLF_L; SLF_R; UF_L; SLF_ (temporal part) L; SLF_(temporal part) R |
|  | 5 | 22338 | (20, 41, 3) | 0.010 | ATR_ L; ATR_ R; CST_L; CST_R; CG_L; CG_R; CH_L; CH_R; F_major; F_minor; IFOF_L; IFOF_R; ILF_R; SLF_L; SLF_R; UF_L; UF_R; SLF_ (temporal part) L; SLF_(temporal part) R |
| MD | 1 | 70689 | (-21, 19, 8) | 0.003 | ATR_ L; ATR_ R; CST_L; CST_R; CG_L; CG_R; CH_L; CH_R; F_major; F_minor; IFOF_L; IFOF_R; ILF_L; ILF_R; SLF_L; SLF_R; UF_L; UF_R; SLF_ (temporal part) L; SLF_(temporal part) R |
| AD | 1 | 706 | (9, -9, 34) | 0.036 | CG_L; CG_R; CH_R; F_minor; SLF_R; SLF_(temporal part) R |
|  | 2 | 39955 | (28, -10, 23) | 0.002 | ATR_ L; ATR_ R; CST_L; CST_R; CG_L; CG_R; CH_L; CH_R; F_major; F_minor; IFOF_L; IFOF_R; ILF_L; ILF_R; SLF_L; SLF_R; UF_L; UF_R; SLF_ (temporal part) L; SLF_(temporal part) R |
| RD | 1 | 95 | (-25, -3, -16) | 0.048 | IFOF_L; ILF_L |
|  | 2 | 56744 | (-28, 16, -2) | 0.003 | ATR_ L; ATR_ R; CST_L; CST_R; CG_L; CG_R; CH_L; CH_R; F_major; F_minor; IFOF_L; IFOF_R; ILF_L; ILF_R; SLF_L; SLF_R; UF_L; UF_R; SLF_ (temporal part) L; SLF_(temporal part) R |
| NDI | 1 | 66149 | (-22, 25, 2) | 0.001 | ATR_ L; ATR_ R; CST_L; CST_R; CG_L; CG_R; CH_L; CH_R; F_major; F_minor; IFOF_L; IFOF_R; ILF_L; ILF_R; SLF_L; SLF_R; UF_L; UF_R; SLF_ (temporal part) L; SLF_(temporal part) R |
| ODI | 1 | 1709 | (27, -7, 24) | 0.007 | ATR_ R; CST_L; CST_R; CG_R; F_major; IFOF_R; ILF_R; SLF_R; UF_R; SLF_(temporal part) R |
|  | 2 | 7249 | (-33, -53, 30) | 0.017 | ATR_ L; ATR_ R; CST_L; CG_L; CG_R; CH_L; CH_R; F_major; F_minor; IFOF_L; ILF_L; SLF_L; SLF_R; UF_L; SLF_ (temporal part) L; SLF_(temporal part) R |
| FISO | 1 | 367 | (43, -23, -2) | 0.040 | ILF_R |
|  | 2 | 11054 | (-8, 28, 11) | 0.007 | ATR_ L; ATR_ R; CST_L; CST_R; CG_L; CG_R; CH_L; CH_R; F_major; F_minor; IFOF_L; IFOF_R; ILF_R; SLF_L; SLF_R; UF_R; SLF_ (temporal part) L; SLF_(temporal part) R |

*p*-value^#^, The *p* - value for controlling the family- wise error is less than 0.05.

AD, axial diffusivity; ATR, anterior thalamic radiation; CG, cingulate gyrus; CH, cingulum hippocampus; CST, corticospinal tract; CTN, classic trigeminal neuralgia; DTI, diffusion tensor imaging; F_major, forceps major; F_minor, forceps minor; IFOF, inferior fronto-occipital fasciculus; ILF, inferior longitudinal fasciculus; L, left; NODDI, neurite orientation dispersion and density imaging; R, right; SLF, superior longitudinal fasciculus; TBSS, Tract-based spatial statistics; UF, uncinate fasciculus.

**Table S10. TBSS analysis of CTN patients with right-side pain based on DTI and NODDI.**

| Metrics | Cluster index | Number of voxels | signal peaks (x, y, z) | *p*-value^#^ | White matter tracts |
| --- | --- | --- | --- | --- | --- |
| FA | 1 | 46893 | (-19, 40, 5) | 0.001 | ATR_ L; ATR_ R; CST_L; CST_R; CG_L; CG_R; CH_L; CH_R; F_major; F_minor; IFOF_L; IFOF_R; ILF_L; ILF_R; SLF_L; SLF_R; UF_L; UF_R; SLF_ (temporal part) L; SLF_(temporal part) R |
| MD | 1 | 27731 | (-18, 28, 20) | 0.008 | ATR_ L; ATR_ R; CST_L; CG_L; CH_L; F_major; F_minor; IFOF_L; ILF_L; SLF_L; SLF_R; UF_L; SLF_ (temporal part) L; SLF_(temporal part) R |
|  | 2 | 29389 | (16, 53, 18) | 0.007 | ATR_ L; ATR_ R; CST_L; CST_R; CG_L; CG_R; CH_L; CH_R; F_major; F_minor; IFOF_R; ILF_R; SLF_L; SLF_R; UF_R; SLF_(temporal part) R |
| AD | 1 | 88 | (-24, 2, 19) | 0.048 | ATR_ L; IFOF_L; SLF_L; UF_L; SLF_(temporal part) R |
|  | 2 | 761 | (24, 12, 16) | 0.037 | ATR_ R; CST_L; CST_R; CG_R; F_minor; IFOF_R; SLF_R; UF_R; SLF_(temporal part) R |
| RD | 1 | 61252 | (-25, 31, -6) | 0.001 | ATR_ L; ATR_ R; CST_L; CST_R; CG_L; CG_R; CH_L; CH_R; F_major; F_minor; IFOF_L; IFOF_R; ILF_L; ILF_R; SLF_L; SLF_R; UF_L; UF_R; SLF_ (temporal part) L; SLF_(temporal part) R |
| NDI | 1 | 7636 | (18, 18, 28) | 0.024 | ATR_ L; ATR_ R; CST_L; CST_R; CG_R; CH_L; CH_R; F_major; F_minor; IFOF_R; ILF_R; SLF_R; UF_R; SLF_(temporal part) R |
|  | 2 | 15031 | (-18, 18, 36) | 0.013 | ATR_ L; ATR_ R; CST_L; CG_L; CH_L; CH_R; F_major; F_minor; IFOF_L; ILF_L; SLF_L; SLF_R; UF_L; SLF_ (temporal part) L; SLF_(temporal part) R |
| FISO | 1 | 34551 | (7, 29, 8) | 0.001 | ATR_ L; ATR_ R; CST_L; CST_R; CG_L; CG_R; CH_L; CH_R; F_major; F_minor; IFOF_L; IFOF_R; ILF_L; ILF_R; SLF_L; SLF_R; UF_L; UF_R; SLF_ (temporal part) L; SLF_(temporal part) R |

*p*-value^#^, The *p* - value for controlling the family- wise error is less than 0.05.

AD, axial diffusivity; ATR, anterior thalamic radiation; CG, cingulate gyrus; CH, cingulum hippocampus; CST, corticospinal tract; CTN, classic trigeminal neuralgia; DTI, diffusion tensor imaging; F_major, forceps major; F_minor, forceps minor; IFOF, inferior fronto-occipital fasciculus; ILF, inferior longitudinal fasciculus; L, left; NODDI, neurite orientation dispersion and density imaging; R, right; SLF, superior longitudinal fasciculus; TBSS, Tract-based spatial statistics; UF, uncinate fasciculus.

**Table S11. Associations between altered white matter tracts (identified by TBSS) and neuropsychological scores in left- and right-sided pain patient groups.**

| **Metrics** | **β** | **se** | **t-value** | ***p*-value** | ***p_FDR_*-value** |
| --- | --- | --- | --- | --- | --- |
| **Left-sided pain patients** | |  |  |  |  |
| **Correlation with VAS** | |  |  |  |  |
| TBSS_FA1 | 6.613 | 5.069 | 1.305 | 0.200 | 0.376 |
| TBSS_FA2 | 4.865 | 13.865 | 0.351 | 0.728 | 0.780 |
| TBSS_FA3 | -22.225 | 8.973 | -2.477 | **0.018** | 0.137 |
| TBSS_FA4 | -30.655 | 13.325 | -2.301 | **0.027** | 0.137 |
| TBSS_FA5 | -22.206 | 12.422 | -1.788 | 0.082 | 0.176 |
| TBSS_MD | 27943.377 | 15481.398 | 1.805 | 0.079 | 0.176 |
| TBSS_AD1 | 11026.748 | 9116.517 | 1.210 | 0.234 | 0.386 |
| TBSS_AD2 | 15620.350 | 13929.931 | 1.121 | 0.270 | 0.386 |
| TBSS_RD1 | 25418.855 | 10880.086 | 2.336 | **0.025** | 0.137 |
| TBSS_RD2 | 25649.094 | 13468.259 | 1.904 | 0.065 | 0.176 |
| TBSS_NDI | -14.592 | 7.978 | -1.829 | 0.076 | 0.176 |
| TBSS_ODI1 | 9.324 | 18.773 | 0.497 | 0.622 | 0.756 |
| TBSS_ODI2 | -7.002 | 33.860 | -0.207 | 0.837 | 0.837 |
| TBSS_FISO1 | 3.102 | 6.891 | 0.450 | 0.655 | 0.756 |
| TBSS_FISO2 | 12.357 | 11.348 | 1.089 | 0.283 | 0.386 |
| **Correlation with SAS** | |  |  |  |  |
| TBSS_FA1 | 16.889 | 41.255 | 0.409 | 0.685 | 0.856 |
| TBSS_FA2 | -163.165 | 107.329 | -1.520 | 0.137 | 0.795 |
| TBSS_FA3 | -42.999 | 77.051 | -0.558 | 0.580 | 0.795 |
| TBSS_FA4 | -109.454 | 112.295 | -0.975 | 0.336 | 0.795 |
| TBSS_FA5 | -83.164 | 102.393 | -0.812 | 0.422 | 0.795 |
| TBSS_MD | 74138.083 | 128285.552 | 0.578 | 0.567 | 0.795 |
| TBSS_AD1 | 8719.236 | 74123.508 | 0.118 | 0.907 | 0.907 |
| TBSS_AD2 | 17175.758 | 112934.138 | 0.152 | 0.880 | 0.907 |
| TBSS_RD1 | 110532.208 | 91236.668 | 1.211 | 0.234 | 0.795 |
| TBSS_RD2 | 76832.553 | 111915.625 | 0.687 | 0.497 | 0.795 |
| TBSS_NDI | -36.690 | 66.209 | -0.554 | 0.583 | 0.795 |
| TBSS_ODI1 | 18.965 | 150.134 | 0.126 | 0.900 | 0.907 |
| TBSS_ODI2 | 159.897 | 268.766 | 0.595 | 0.556 | 0.795 |
| TBSS_FISO1 | 45.665 | 54.563 | 0.837 | 0.408 | 0.795 |
| TBSS_FISO2 | 68.269 | 91.232 | 0.748 | 0.459 | 0.795 |
| **Correlation with SDS** | |  |  |  |  |
| TBSS_FA1 | 14.405 | 46.377 | 0.311 | 0.758 | 0.934 |
| TBSS_FA2 | -10.332 | 124.332 | -0.083 | 0.934 | 0.934 |
| TBSS_FA3 | 34.924 | 86.710 | 0.403 | 0.689 | 0.934 |
| TBSS_FA4 | -26.921 | 127.687 | -0.211 | 0.834 | 0.934 |
| TBSS_FA5 | 23.085 | 115.977 | 0.199 | 0.843 | 0.934 |
| TBSS_MD | -67019.732 | 144305.517 | -0.464 | 0.645 | 0.934 |
| TBSS_AD1 | -85461.647 | 82032.615 | -1.042 | 0.304 | 0.761 |
| TBSS_AD2 | -134997.974 | 124859.817 | -1.081 | 0.287 | 0.761 |
| TBSS_RD1 | 11604.639 | 104512.790 | 0.111 | 0.912 | 0.934 |
| TBSS_RD2 | -46279.427 | 126270.967 | -0.367 | 0.716 | 0.934 |
| TBSS_NDI | 15.549 | 74.628 | 0.208 | 0.836 | 0.934 |
| TBSS_ODI1 | 180.390 | 165.943 | 1.087 | 0.284 | 0.761 |
| TBSS_ODI2 | 357.739 | 297.399 | 1.203 | 0.237 | 0.761 |
| TBSS_FISO1 | 94.860 | 59.816 | 1.586 | 0.122 | 0.761 |
| TBSS_FISO2 | 186.772 | 98.448 | 1.897 | 0.066 | 0.761 |
| **Right-sided pain patients** | |  |  |  |  |
| **Correlation with VAS** | |  |  |  |  |
| TBSS_FA | -121.955 | 92.999 | -1.311 | 0.195 | 0.254 |
| TBSS_MD1 | 128739.675 | 98804.876 | 1.303 | 0.198 | 0.254 |
| TBSS_MD2 | 142883.387 | 99488.019 | 1.436 | 0.157 | 0.254 |
| TBSS_AD1 | 60092.431 | 42984.903 | 1.398 | 0.168 | 0.254 |
| TBSS_AD2 | 55487.518 | 32241.127 | 1.721 | 0.091 | 0.254 |
| TBSS_RD | 130669.060 | 96240.127 | 1.358 | 0.180 | 0.254 |
| TBSS_NDI4 | -13.907 | 46.303 | -0.300 | 0.765 | 0.765 |
| TBSS_NDI3 | -131.952 | 46.537 | -2.835 | **0.006** | 0.057 |
| TBSS_FISO | 20.931 | 59.548 | 0.351 | 0.727 | 0.765 |
| **Correlation with SAS** | |  |  |  |  |
| TBSS_FA | -121.955 | 92.999 | -1.311 | 0.195 | 0.254 |
| TBSS_MD1 | 128739.675 | 98804.876 | 1.303 | 0.198 | 0.254 |
| TBSS_MD2 | 142883.387 | 99488.019 | 1.436 | 0.157 | 0.254 |
| TBSS_AD1 | 60092.431 | 42984.903 | 1.398 | 0.168 | 0.254 |
| TBSS_AD2 | 55487.518 | 32241.127 | 1.721 | 0.091 | 0.254 |
| TBSS_RD | 130669.060 | 96240.127 | 1.358 | 0.180 | 0.254 |
| TBSS_NDI4 | -13.907 | 46.303 | -0.300 | 0.765 | 0.765 |
| TBSS_NDI3 | -131.952 | 46.537 | -2.835 | **0.006** | 0.057 |
| TBSS_FISO | 20.931 | 59.548 | 0.351 | 0.727 | 0.765 |
| **Correlation with SDS** | |  |  |  |  |
| TBSS_FA | -41.426 | 105.149 | -0.394 | 0.695 | 0.894 |
| TBSS_MD1 | 54084.537 | 111857.939 | 0.484 | 0.631 | 0.894 |
| TBSS_MD2 | 60923.653 | 112926.018 | 0.540 | 0.592 | 0.894 |
| TBSS_AD1 | 3034.673 | 48686.650 | 0.062 | 0.951 | 0.951 |
| TBSS_AD2 | 23404.061 | 36697.999 | 0.638 | 0.526 | 0.894 |
| TBSS_RD | 49306.432 | 109012.090 | 0.452 | 0.653 | 0.894 |
| TBSS_NDI4 | 8.272 | 51.791 | 0.160 | 0.874 | 0.951 |
| TBSS_NDI3 | -119.379 | 53.267 | -2.241 | **0.029** | 0.262 |
| TBSS_FISO | 32.400 | 66.196 | 0.489 | 0.626 | 0.894 |

β: Standardized beta coefficient of separate linear regression analysis; *p_FDR_*-value, false discovery rate -corrected *p* value.

AD, axial diffusivity; FA, fractional anisotropy; FISO, fraction of isotropic diffusion; MD, mean diffusivity; NDI, neurite density index; ODI, orientation dispersion index; RD, radial diffusivity; SAS, self-rating anxiety scale; SDS, self-rating depression scale; TBSS, Tract-based spatial statistics; VAS, visual analogue scale.

**Table S12. Results of mediation analyses.**

|  | effect | se | LLCI | ULCI |
| --- | --- | --- | --- | --- |
| Left-sided pain patients |  |  |  |  |
| FA- left DTI-ALPS index-TBSS_FA1 |  |  |  |  |
| Total effect | 0.143 | 0.142 | -0.144 | 0.430 |
| Direct effect | 0.146 | 0.145 | -0.149 | 0.441 |
| Indirect effect | -0.003 | 0.031 | -0.070 | 0.064 |
| FA- left DTI-ALPS index-TBSS_FA2 |  |  |  |  |
| Total effect | 0.033 | 0.053 | -0.075 | 0.141 |
| Direct effect | 0.022 | 0.053 | -0.087 | 0.130 |
| Indirect effect | 0.011 | 0.018 | -0.016 | 0.054 |
| FA- left DTI-ALPS index-TBSS_FA3 |  |  |  |  |
| Total effect | -0.013 | 0.077 | -0.169 | 0.143 |
| Direct effect | -0.033 | 0.076 | -0.187 | 0.122 |
| Indirect effect | 0.020 | 0.030 | -0.018 | 0.098 |
| FA- left DTI-ALPS index-TBSS_FA4 |  |  |  |  |
| Total effect | 0.045 | 0.052 | -0.061 | 0.150 |
| Direct effect | 0.034 | 0.052 | -0.072 | 0.140 |
| Indirect effect | 0.011 | 0.016 | -0.010 | 0.053 |
| FA- left DTI-ALPS index-TBSS_FA5 |  |  |  |  |
| Total effect | 0.043 | 0.057 | -0.073 | 0.159 |
| Direct effect | 0.030 | 0.057 | -0.087 | 0.146 |
| Indirect effect | 0.013 | 0.018 | -0.013 | 0.060 |
| MD- left DTI-ALPS index-TBSS_MD |  |  |  |  |
| Total effect | -0.008 | 0.020 | -0.045 | 0.036 |
| Direct effect | -0.002 | 0.020 | -0.043 | 0.039 |
| Indirect effect | -0.008 | 0.006 | -0.021 | 0.005 |
| AD- left DTI-ALPS index-TBSS_AD1 |  |  |  |  |
| Total effect | 0.003 | 0.025 | -0.048 | 0.053 |
| Direct effect | 0.001 | 0.0260 | -0.052 | 0.054 |
| Indirect effect | 0.002 | 0.011 | -0.026 | 0.019 |
| AD- left DTI-ALPS index-TBSS_AD2 |  |  |  |  |
| Total effect | 0.013 | 0.016 | -0.020 | 0.045 |
| Direct effect | 0.015 | 0.017 | -0.019 | 0.049 |
| Indirect effect | -0.002 | 0.008 | -0.025 | 0.007 |
| RD- left DTI-ALPS index-TBSS_RD1 |  |  |  |  |
| Total effect | -0.022 | 0.026 | -0.074 | 0.031 |
| Direct effect | -0.021 | 0.026 | -0.073 | 0.032 |
| Indirect effect | -0.001 | 0.006 | -0.015 | 0.011 |
| RD- left DTI-ALPS index-TBSS_RD2 |  |  |  |  |
| Total effect | -0.014 | 0.022 | -0.057 | 0.030 |
| Direct effect | -0.012 | 0.021 | -0.056 | 0.031 |
| Indirect effect | -0.001 | 0.006 | -0.017 | 0.010 |
| NDI- left DTI-ALPS index-TBSS_NDI |  |  |  |  |
| Total effect | 0.041 | 0.035 | -0.029 | 0.111 |
| Direct effect | 0.037 | 0.035 | -0.035 | 0.108 |
| Indirect effect | 0.004 | 0.010 | -0.021 | 0.021 |
| ODI- left DTI-ALPS index-TBSS_ODI1 |  |  |  |  |
| Total effect | 0.001 | 0.021 | -0.041 | 0.044 |
| Direct effect | 0.007 | 0.022 | -0.038 | 0.051 |
| Indirect effect | -0.006 | 0.008 | -0.026 | 0.006 |
| ODI- left DTI-ALPS index-TBSS_ODI2 |  |  |  |  |
| Total effect | 0.001 | 0.012 | -0.023 | 0.024 |
| Direct effect | -0.001 | 0.012 | -0.026 | 0.024 |
| Indirect effect | 0.002 | 0.006 | -0.013 | 0.013 |
| FISO- left DTI-ALPS index-TBSS_FISO1 |  |  |  |  |
| Total effect | 0.130 | 0.037 | 0.055 | 0.206 |
| Direct effect | 0.096 | 0.039 | 0.017 | 0.175 |
| Indirect effect | 0.034 | 0.028 | -0.012 | 0.096 |
| FISO- left DTI-ALPS index-TBSS_FISO2 |  |  |  |  |
| Total effect | 0.053 | 0.024 | 0.003 | 0.102 |
| Direct effect | 0.053 | 0.027 | -0.002 | 0.108 |
| Indirect effect | -0.000 | 0.015 | -0.036 | 0.026 |
| Right-sided pain patients |  |  |  |  |
| FA- right DTI-ALPS index-TBSS_FA |  |  |  |  |
| Total effect | -0.018 | 0.037 | -0.093 | 0.056 |
| Direct effect | -0.015 | 0.036 | -0.087 | 0.057 |
| Indirect effect | -0.003 | 0.010 | -0.026 | 0.017 |
| MD- right DTI-ALPS index-TBSS_MD1 |  |  |  |  |
| Total effect | 0.056 | 0.024 | 0.008 | 0.104 |
| Direct effect | 0.050 | 0.024 | 0.001 | 0.098 |
| Indirect effect | 0.006 | 0.007 | -0.004 | 0.023 |
| MD- right DTI-ALPS index-TBSS_MD2 |  |  |  |  |
| Total effect | 0.053 | 0.024 | 0.005 | 0.101 |
| Direct effect | 0.046 | 0.024 | -0.002 | 0.094 |
| Indirect effect | 0.007 | 0.008 | -0.004 | 0.026 |
| AD- right DTI-ALPS index-TBSS_AD1 |  |  |  |  |
| Total effect | -0.019 | 0.037 | -0.092 | 0.055 |
| Direct effect | -0.028 | 0.036 | -0.100 | 0.045 |
| Indirect effect | 0.009 | 0.011 | -0.008 | 0.035 |
| AD- right DTI-ALPS index-TBSS_AD2 |  |  |  |  |
| Total effect | 0.029 | 0.049 | -0.068 | 0.126 |
| Direct effect | 0.024 | 0.049 | -0.075 | 0.122 |
| Indirect effect | 0.006 | 0.010 | -0.013 | 0.030 |
| RD- right DTI-ALPS index-TBSS_RD |  |  |  |  |
| Total effect | 0.039 | 0.022 | -0.005 | 0.082 |
| Direct effect | 0.032 | 0.022 | -0.011 | 0.075 |
| Indirect effect | 0.007 | 0.007 | -0.005 | 0.024 |
| NDI- right DTI-ALPS index-TBSS_NDI1 |  |  |  |  |
| Total effect | 0.030 | 0.029 | -0.029 | 0.088 |
| Direct effect | 0.018 | 0.030 | -0.043 | 0.078 |
| Indirect effect | 0.012 | 0.010 | -0.007 | 0.033 |
| NDI- right DTI-ALPS index-TBSS_NDI2 |  |  |  |  |
| Total effect | 0.038 | 0.031 | 0.025 | 0.100 |
| Direct effect | 0.014 | 0.032 | -0.049 | 0.077 |
| Indirect effect | 0.023 | 0.013 | 0.002 | 0.052 |
| FISO- right DTI-ALPS index-TBSS_FISO |  |  |  |  |
| Total effect | 0.001 | 0.032 | -0.064 | 0.065 |
| Direct effect | 0.005 | 0.031 | -0.056 | 0.067 |
| Indirect effect | -0.005 | 0.012 | -0.026 | 0.023 |

AD, axial diffusivity; DTI-ALPS, diffusion tensor imaging along the perivascular space; FA, fractional anisotropy; FISO, fraction of isotropic diffusion; LLCI, lower limit of the confidence interval; MD, mean diffusivity; NDI, neurite density index; ODI, orientation dispersion index; RD, radial diffusivity; TBSS, Tract-based spatial statistics; ULCI, upper limit of the confidence interval.

**Table S13.Diagnostic performance of individual imaging metrics for discriminating CTN patients from HCs using 5-fold cross-validation.**

| **Metrics** | **AUC** | **LLCI** | **ULCI** | **Sensitivity** | **Specificity** | ***p*-value** |
| --- | --- | --- | --- | --- | --- | --- |
| **Peripheral diffusion metrics** | | | | | | |
| FA | 0.685 | 0.610 | 0.760 | 0.683 | 0.621 | < 0.001 |
| MD | 0.542 | 0.397 | 0.562 | 0.154 | 0.908 | 0.625 |
| AD | 0.527 | 0.386 | 0.559 | 0.356 | 0.690 | 0.446 |
| RD | 0.531 | 0.333 | 0.595 | 0.077 | 0.977 | 0.037 |
| NDI | 0.612 | 0.531 | 0.692 | 0.471 | 0.793 | 0.006 |
| ODI | 0.563 | 0.426 | 0.591 | 0.654 | 0.448 | 0.844 |
| FISO | 0.647 | 0.519 | 0.681 | 0.769 | 0.414 | 0.016 |
| DTI-ALPS indexes | |  |  |  |  |  |
| DTI-ALPS indexes of the affected side | 0.647 | 0.557 | 0.714 | 0.683 | 0.552 | 0.001 |
| total DTI-ALPS indexes | 0.608 | 0.492 | 0.655 | 0.471 | 0.690 | 0.078 |
| **Diffusion metrics within significant TBSS clusters of left-sided pain patients** | | | | | | |
| TBSS_FA1 | 0.690 | 0.574 | 0.807 | 0.595 | 0.786 | 0.001 |
| TBSS_FA2 | 0.783 | 0.685 | 0.880 | 0.810 | 0.643 | < 0.001 |
| TBSS_FA3 | 0.748 | 0.646 | 0.851 | 0.905 | 0.452 | < 0.001 |
| TBSS_FA4 | 0.781 | 0.683 | 0.879 | 0.857 | 0.619 | < 0.001 |
| TBSS_FA5 | 0.759 | 0.655 | 0.863 | 0.786 | 0.643 | < 0.001 |
| TBSS_MD | 0.739 | 0.631 | 0.847 | 0.643 | 0.810 | < 0.001 |
| TBSS_AD1 | 0.779 | 0.681 | 0.876 | 0.690 | 0.762 | < 0.001 |
| TBSS_AD2 | 0.715 | 0.605 | 0.824 | 0.929 | 0.476 | < 0.001 |
| TBSS_RD1 | 0.764 | 0.659 | 0.870 | 0.929 | 0.619 | < 0.001 |
| TBSS_RD2 | 0.739 | 0.632 | 0.846 | 0.810 | 0.619 | < 0.001 |
| TBSS_NDI | 0.713 | 0.600 | 0.826 | 0.762 | 0.667 | < 0.001 |
| TBSS_ODI1 | 0.709 | 0.598 | 0.819 | 0.690 | 0.714 | < 0.001 |
| TBSS_ODI2 | 0.811 | 0.798 | 0.944 | 0.714 | 0.881 | < 0.001 |
| TBSS_FISO1 | 0.750 | 0.645 | 0.855 | 0.643 | 0.810 | < 0.001 |
| TBSS_FISO2 | 0.771 | 0.671 | 0.871 | 0.905 | 0.524 | < 0.001 |
| **Diffusion metrics within significant TBSS clusters of right-sided pain patients** | | | | | | |
| TBSS_FA | 0.744 | 0.657 | 0.831 | 0.855 | 0.597 | < 0.001 |
| TBSS_MD1 | 0.651 | 0.554 | 0.748 | 0.855 | 0.435 | 0.002 |
| TBSS_MD2 | 0.640 | 0.541 | 0.739 | 0.677 | 0.613 | 0.006 |
| TBSS_AD1 | 0.638 | 0.539 | 0.737 | 0.855 | 0.468 | 0.006 |
| TBSS_AD2 | 0.631 | 0.532 | 0.729 | 0.500 | 0.726 | 0.009 |
| TBSS_RD | 0.682 | 0.587 | 0.777 | 0.871 | 0.484 | < 0.001 |
| TBSS_NDI4 | 0.643 | 0.545 | 0.741 | 0.758 | 0.532 | 0.004 |
| TBSS_NDI3 | 0.645 | 0.548 | 0.742 | 0.806 | 0.435 | 0.003 |
| TBSS_FISO | 0.739 | 0.649 | 0.829 | 0.903 | 0.581 | < 0.001 |

AD, axial diffusivity; AUC, area under the curve; CTN, classic trigeminal neuralgia; DTI-ALPS, diffusion tensor imaging along the perivascular space; FA, fractional anisotropy; FISO, fraction of isotropic diffusion; HCs, healthy controls; LLCI, lower limit of the confidence interval; MD, mean diffusivity; NDI, neurite density index; ODI, orientation dispersion index; RD, radial diffusivity; TBSS, Tract-based spatial statistics; ULCI, upper limit of the confidence interval.

**Table S14. Diagnostic performance of multivariable models for discriminating CTN patients from HCs using 5-fold cross-validation.**

| **Metrics** | **AUC** | **LLCI** | **ULCI** | **Sensitivity** | **Specificity** | ***p*-value** |
| --- | --- | --- | --- | --- | --- | --- |
| **Left-sided pain patients** | | |  |  |  |  |
| FA | 0.796 | 0.701 | 0.891 | 0.881 | 0.595 | < 0.001 |
| MD | 0.726 | 0.616 | 0.835 | 0.667 | 0.714 | < 0.001 |
| AD | 0.793 | 0.698 | 0.889 | 0.619 | 0.881 | < 0.001 |
| RD | 0.747 | 0.637 | 0.857 | 0.762 | 0.738 | < 0.001 |
| NDI | 0.692 | 0.579 | 0.805 | 0.833 | 0.500 | 0.001 |
| ODI | 0.822 | 0.734 | 0.910 | 0.690 | 0.833 | < 0.001 |
| FISO | 0.775 | 0.673 | 0.877 | 0.714 | 0.762 | < 0.001 |
| **Right-sided pain patients** | | |  |  |  |  |
| FA | 0.811 | 0.755 | 0.867 | 0.855 | 0.677 | < 0.001 |
| MD | 0.719 | 0.629 | 0.808 | 0.774 | 0.565 | < 0.001 |
| AD | 0.784 | 0.704 | 0.863 | 0.629 | 0.806 | < 0.001 |
| RD | 0.732 | 0.642 | 0.821 | 0.871 | 0.565 | < 0.001 |
| NDI | 0.717 | 0.627 | 0.807 | 0.694 | 0.661 | < 0.001 |
| FISO | 0.780 | 0.700 | 0.860 | 0.613 | 0.823 | < 0.001 |

AD, axial diffusivity; AUC, area under the curve; CTN, classic trigeminal neuralgia; FA, fractional anisotropy; FISO, fraction of isotropic diffusion; HCs, healthy controls; LLCI, lower limit of the confidence interval; MD, mean diffusivity; NDI, neurite density index; ODI, orientation dispersion index; RD, radial diffusivity; ULCI, upper limit of the confidence interval.
